# Acclimation of cadmium-induced genotoxicity and oxidative stress in mung bean seedlings by priming effect of phytohormones and proline

**DOI:** 10.1371/journal.pone.0257924

**Published:** 2021-09-29

**Authors:** Meher Hassan, Muhammad Israr, Simeen Mansoor, Syeda Amna Hussain, Faiza Basheer, Azizullah Azizullah, Shafiq Ur Rehman

**Affiliations:** 1 Department of Genetics, University of Karachi, Sindh, Pakistan; 2 Department of Biology, The University of Haripur, Haripur, Khyber Pakhtunkhwa, Pakistan; 3 College of Life Science, Hebei Normal University, Shijiazhuang, Hebei, PR China; 4 Institute of Chemical Sciences, University of Peshawar, Peshawar, Khyber Pakhtunkhwa, Pakistan; 5 Department of Zoology, Women University Mardan, Mardan, Khyber Pakhtunkhwa Pakistan; Government College University Faisalabad, PAKISTAN

## Abstract

In this research, eight local mung bean (*Vigna radiata*) varieties were analyzed for their performance against two levels of CdCl_2_ solution (0.3 and 0.5 mM) alone and priming with gibberellic acid (GA_3_) (100 μM), salicylic acid (SA) (50 μM) and proline (5 mM) solution prior to Cd exposure. Mung bean seedlings were analyzed for disturbance in cytological, morphological, biochemical and enzymatic parameters under cadmium stress. For cytological studies, 48 h grown mung bean seedlings root tips were used to prepare slides and studied for percent mitotic index (MI%) and to calculate percent C-mitosis, laggard, sticky and fragmented chromosomes, pictures were captured by a Nikon camera (DS-Fi 1 Japan) attached with a microscope. One-week grown mung seedlings were studied for growth traits, malondialdehyde (MDA), protein, proline and antioxidant enzymes. ANOVA and DMR test of this research revealed that all the tested mung bean varieties and treatments were significantly different regarding mitotic index and number of chromosomal aberrations. Both the Cd treatments exhibited increased total chromosomal aberrations with different types and a maximum decrease in MI%. In pretreated samples, GA_3_, SA and proline serve as mitigating agents that reduce mutagenic effects of Cd in mung bean by increasing MI% and decreasing chromosomal aberrations as compared to non-pretreated samples. Both the Cd treatments showed a decrease in all growth traits. Total proteins were also found to be significantly reduced in a dose-dependent manner in all genotypes. Cd treatment increased the activities of all antioxidant enzymes tested. Cd caused oxidative damage as indicated by elevated levels of MDA content in treated samples in comparison to control. Proline content levels were also high in Cd treated seedlings indicating stress. Results demonstrated that pretreatment with phytohormones and proline before Cd were found to improve all morphological parameters, by altering antioxidant enzymes activities along with a decrease in MDA and proline contents as well. It was further noticed that the performance of GA_3_ was better at 0.3 mM Cd treatment while SA was found to be a good mitigating agent at 0.5 mM Cd stress in all tested mung bean varieties. This research concluded less deleterious effects of Cd on AZRI-2006 while more sensitivity to NM-51 towards Cd. Priming with phytohormones and proline is a user-friendly, economical, and simple mitigation strategy to reduce Cd toxicity in plants and get better yield from contaminated lands.

## Introduction

Some metals are found naturally in the earth’s crust and anthropogenic events tend to increase levels of other metals that are contaminating our ecosystem [1]. As micronutrients, these metals have an important role in plant metabolism, but these can be highly toxic for all plants if stored in exorbitant amounts [2]. Plant roots can absorb these metals directly from soil and water or indirectly by foliar application, causing higher accumulation in different plant parts [3]. Poor farming practices and disposal of untreated industrial and urban waste are contaminating agricultural soils in developing countries [4]. The augmentation of heavy metals in the water and soil from various resources can reach us by food chain that is responsible for certain physiological and biochemical anomalies with serious chronic health problems that include itai-itai disease, different cancers, and kidney and liver disorders [5]. Cadmium (Cd) is among the most hazardous mobile element found abundantly in soil, causing growth reduction by altering different processes including photosynthesis, mineral transport, protein and cell membrane damage. They may also cause a disturbance in cell division along with structural alterations in plants, inactivation of enzymes and hormonal imbalance thus reducing crop productivity [6]. Cd interrupts the normal cell cycle thus causing chromosomal abnormalities in cells [7]. Aberrations were resulted due to direct damage to DNA and interference of Cd ions in the transcription and translation process that hinders the synthesis of DNA apparatus. Cd might also disturb enzymes involves in the DNA repair mechanism, either by modifying their structure or by reducing the levels of transcriptomes that can cause aberrations [8]. Studies revealed that Cd treatment in plants can cause growth reduction related to inhibition in mitotic index, by inducing chromosomal and nuclear aberrations in the root meristems [9–11]. ROS production/accumulation in plants is accountable for generating oxidative stress under Cd stress. Plasma membrane permeability was transformed in oxidative stress by restraining ATPase activities, which will assist in ionic homeostasis throughout the cells [12, 13].

Therefore, adopting mitigating strategies is important in reducing the Cd accumulation in plants specifically in edible parts. Remediation techniques have been used previously which were costly, time-consuming and not easy to perform with major side effects as well [14].

Gibberellic acid (GA_3_) is an endogenous phytohormone that belongs to terpenoids and growth regulator which stimulates hydrolytic enzymes and is helpful in the germination process of seeds [15, 16]. Salicylic acid (SA) is an endogenous signaling molecule and plant growth regulators have major roles in various abiotic stresses like heavy metals in different crops [17]. Under harsh conditions, plant metabolites like amino acids accumulate in different parts; many amino acids like proline have major roles as protein precursors and building blocks, helpful in plant metabolism and development. They act as major osmolytes, a metal chelator, an antioxidant and signaling molecule during different kinds of abiotic stress [9, 18].

Mung bean (*Vigna radiata* (L.) Wilczek) has been selected as research material as it is very popular in Asia but sensitive to Cd. It is a short-duration, bi-annual, warm seasoned leguminous crop, widely grown for edible purposes. Mung bean has high levels of proteins, folate and iron [19]. It is also a good atmospheric nitrogen fixer at or in the soil that assists in improving soil fertility thus has a major role in the intercropping system [20], thus can reduce the use of expensive fertilizers. Limited research about Cd toxicity in mung beans resulted in slower progress in crop improvement. Thus, one of the reasons for conducting present research was to examine the impact of Cd with or without prior treatment of phytohormones and proline on mung bean genotypes. Morphological parameters such as overall seedling length, fresh and dry weight, relative water content and certain biochemical changes including protein were studied. Stress-induced malondialdehyde (MDA) generation was taken under consideration as a marker for Cd toxicity. Antioxidant defensive molecules such as proline and antioxidant enzymes were analyzed. Furthermore, the mitigating effect of phytohormones and proline on acclimatizing Cd toxicity in different local mung bean germplasms based on mitotic index and percent chromosomal aberrations were analyzed. These strategies may be applied by mung bean farmers to make use of metal-polluted lands for cultivation and to improve mung bean growth and yield under cadmium stress.

## Material and methods

### Plant material

Seeds of mung bean varieties (NM 2006, NM 19–19, NM 2011, NM 20–21, NM 121–123, AZRI-2006, NM 13–1 and NM-51) were obtained from National Agricultural Research Center (NARC), Islamabad, Pakistan.

### Experimental condition

Healthy and uniformed-sized seeds were sterilized by using 1% sodium hypochlorite for 5 minutes, later washed thrice using distilled water (D/W). Priming of seeds was done with D/W (non-pretreated), and GA_3_ (100 μM), SA (50 μM), and proline (5 mM) solutions (pretreated) for 12 h in 50 ml beakers separately. The experiment was set in CRD with 3 replications. Pretreated and non-pretreated twenty seeds were germinated in each petri dish (6 inches) layered with filter paper moistened with 5 ml of D/W for 24 h at 30°C. Germinated pretreated and non-pretreated seedlings were later given the treatment of 0.3 mM and 0.5 mM CdCl_2_ for 1 week.

### Slides preparation

For slides preparation, three seedlings were selected randomly from all replications at 48 h. Two days old mung bean root tips having 2 cm length were cut with a sharp scalpel. Excess amount of Cd was removed by washing with D/W and placed in Farmer’s fixative (alcohol-acetic acid 3:1) for one day. Fixed root tips were then stained in 1.8% aceto-orcein for 7 days. Hydrochloric acid (1M) was used for 9 min at 60°C to hydrolyze root tips later tapped in 45% glacial acetic acid on clean glass slides [21, 22].

### Calculation for mitotic index and chromosomal aberrations

Approximately 500 cells were examined for mitotic divisions and chromosomal aberrations for each slide at an oil immersion lens i.e. 100 X. Photographs were taken by Nikon camera (DS-Fi 1 Japan) attached with a microscope.

Mitotic index and chromosomal aberrations were calculated by the following formula

MI%=(Dividingcells/Totalcells)x100

Aberrationpercentage=(Aberrantcells/Totalcells)x100

### Morphological traits

At the termination of experiment, seedlings were collected and stored at 4°C to proceed further. Seedling length (8 days old) was measured in centimeters (cm). Fresh weight (FW) was weighed in grams (g) and Dry weight (DW) was calculated by desiccating seedlings in an incubator for 24 h at 70°C. Water content was calculated by the following formula [23].


RWC%=Freshweight-Dryweight/Freshweightx100

### Protein and enzymatic analysis

Seedlings (0.2 g) were crushed in 50 mM sodium phosphate buffer (pH 7.0) (containing 1% PVP & 0.2 mM ascorbic acid) in an ice-cold mortar and pestle and centrifuge at 10,000 rpm for 30 min in cold conditions [24]. The supernatant was saved at 4°C for further enzymatic analysis and protein estimation [25] using bovine serum albumin (BSA) as a standard.

### Antioxidant enzymes estimation

Ascorbate peroxidase (APX) reaction mixture contained 450 μL enzyme, 50 mM sodium phosphate buffer (pH 7.0), 0.1 mM EDTA and 0.5 mM ascorbic acid. Initiation of reaction was done by adding 0.1 mM H_2_O_2_ and taking absorbance at 290 nm for 2 min at the interval of 15 sec. The specific activity of APX was calculated by using a formula with E.C 2.8 mM^–1^ cm^–1^ [26].

The Reaction mixture for catalase (CAT) consisted of 50 mM potassium phosphate buffer (pH 7.0) and 50 μL enzyme. Enzyme activity was initiated by adding 12.5 mM H_2_O_2_ and followed degradation at 240 nm for 2 min (E.C 40 mM^–1^ cm^–1^) for calculating specific activity [27].

Guaiacol peroxidase (GPX) activity was determined by taking extracted supernatant (50 μL) added with 150 mM sodium phosphate buffer (pH 5.6), 100 mM guaiacol solution and 176 mM H_2_O_2_. Absorbance was taken at 470 nm against the reagent blank. Calculation for the specific activity of GPX was done as followed with E.C 26.6 mM^–1^ cm^–1^ [28].

Superoxide dismutase (SOD) activity was assayed by measuring enzyme capability for photochemical reduction of nitro blue tetrazolium (NBT). Reaction mixture consisted of 100 mM potassium phosphate buffer (pH 7.5), 1500 mM sodium carbonate, 200 mM methionine, 2.25 mM NBT, 3 mM EDTA, 1 mL distilled water and 150 μL of enzyme extract incubated at dark for 8 min and 60 μM riboflavin was added at the end. Vortex the reaction mixture and place 30 cm below the light source consisting of 40 W fluorescent lamps for half an hour. Tubes kept in dark were served as a blank, while the control tube was without the enzyme and kept in the light. The absorbance was measured at 560 nm. One unit of activity is the amount of enzymes required to inhibit 50% initial reduction of NBT under light [29].

### MDA contents estimation

Lipid peroxidation was determined by estimating the malondialdehyde (MDA) content. Harvested seedlings were extracted in 5% TCA in cold conditions. Homogenate was then spun at 12000 rpm for 15 min. For estimation of MDA, 500 μL homogenate was added in 0.5% TBA in a 20% TCA solution. The reaction mixture was heated at 95°C for 25 min and then the reaction was stopped on ice. Optical density was recorded at 532 and 600 nm for subtracting non-specific absorbance. MDA content was calculated with E.C of 155 mM^–1^ cm^–1^ [30].

### Proline contents estimation

To estimate free proline contents, seedlings were crushed in 3% sulphosalicylic acid then centrifuged at 10000 rpm for 10 min. An equal volume of extracted material, ninhydrin reagent (1.25 g of ninhydrin in 30 mL of glacial acetic acid and 20 mL of 6 M orthophosphoric acid) and glacial acetic acid were mixed in a test tube. Incubation at 100°C on boiling water bath for 1 h was done; ice was immediately added to stop the reaction. Toluene was added to the solution followed by thorough mixing. The chromophore toluene layer was aspirated from the aqueous phase whose OD was read at 520 nm. Toluene will serve as a blank [31]. Proline was calculated according to the formula mention below

Proline=[(μgproline/mL×mLtoluene)/115.5μg/μmol]/[(gsample)/5]

### Statistical analysis

A lab experiment was set in petri dishes/treatment/variety as a completely randomized design (CRD) with three replications. All data were subjected to ANOVA by using Computer Program IBM SPSS version 20. DMRT at the P ≤ 0.05 level of significance was performed. Similar alphabets in bar graphs showed non-significant differences between the treatments for each variety. Vertical bars on graphs represent standard errors (n = 3) [32].

## Results

### Cytological analysis

Highly significant differences among all eight tested varieties and treatments along with a significant interaction between both the factors for mitotic index and chromosomal aberrations were depicted as shown in Table 1.

**Table 1:** Mean sum of squares for MI% and chromosomal aberrations from mung bean root tips under Cd alone and priming with phytohormones and proline before Cd stress.

SOV	Df	MS					
		MI%	Stickiness	Laggard	C-mitosis	Fragmentation	Total Aber
Varieties	7	1202.791**	4.995**	6.802**	8.103**	5.608**	80.868**
Treatment	8	803.781**	70.737**	31.074**	111.001**	93.147**	1142.598**
V×T	56	33.227**	0.749**	1.313**	0.995**	2.05**	7.884**
Error	144	2.329**	0.116**	0.083**	0.162**	0.144**	0.329**

### Effect of Cd on mitotic index

**Fig 1:**
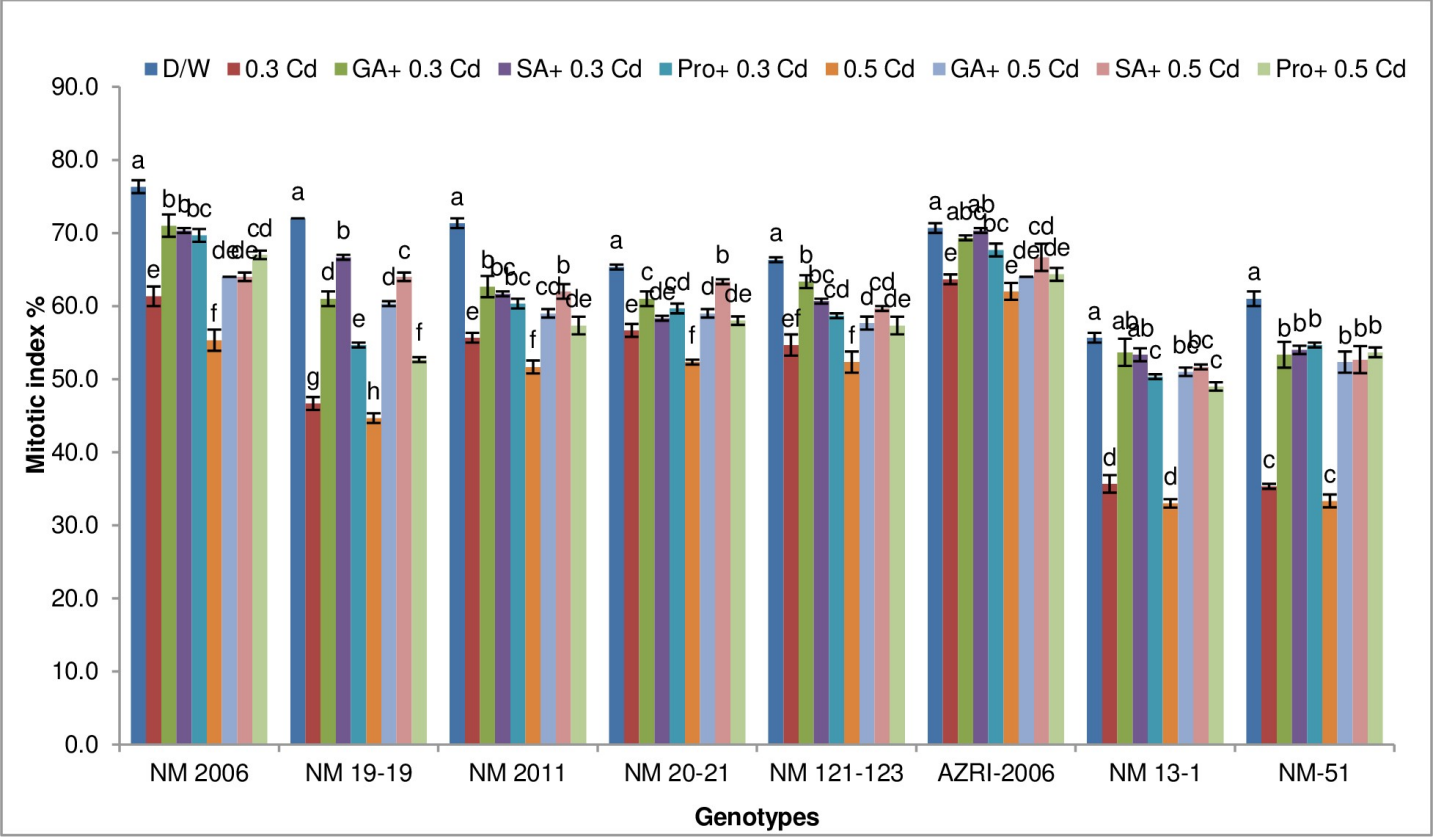
Mean mitotic index of mung bean root tips under Cd treatments alone and priming with phytohormones and proline prior to Cd stress. Different alphabets exhibit significant differences among the treatments for each variety. Vertical bars shows standard error with n = 3 at the P ≤ 0.05 level of significance.

Results of this research indicated that different concentrations of Cd tend to reduce cell division of root cells, indicated by lower values of the mitotic index as compared to control (Fig 1).

Highest but variable values for MI % in control (D/W) for all mung bean varieties among all treatments were noticed with given hierarchal pattern NM 2006 > NM 19–19 > NM 2011 > AZRI-2006 > NM 121–123 > NM 20–21 > NM-51 > NM 13–1.

During Cd treatment, MI% decreased gradually as Cd concentration was increased. AZRI-2006 performed very well by inhibiting MI% only by 10% at 0.3 mM Cd and 12% at 0.5 mM Cd whereas the highest inhibition was noticed in NM-51 which was 42% at 0.3 mM Cd and 45% at 0.5 mM Cd. However, priming with GA_3_, SA and proline prior to Cd tends to ameliorate the toxic effect of Cd as indicated in S1 Fig. It was noticed that priming with GA_3_ was better for 0.3 mM Cd for most of the varieties. However, SA gave a better amelioration effect when applied prior to 0.5 mM Cd for all varieties except for NM 2006. For AZRI-2006, SA pretreatment was best and exhibited 0.5% inhibition at 0.3 mM Cd whereas 6% inhibition in MI% for 0.5 mM Cd. However, for NM-51, priming with proline was more efficient than GA_3_ and SA in reducing the clastogenic effect of Cd, showing 10% inhibition at 0.3 mM and 12% for 0.5 mM Cd treatment.

### Increase in chromosomal aberrations under Cd stress

**Fig 2:**
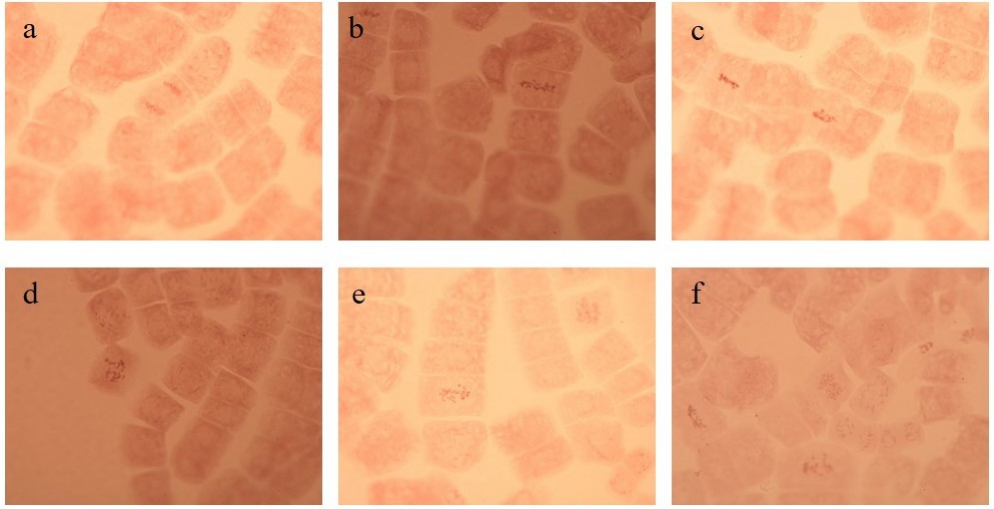
Pictorial demonstration of various normal and aberrated chromosomes observed in root tips of mung beans grown under different treatments (a) Normal Anaphase (b) Normal Metaphase (c) Sticky chromosomes (d) Laggard chromosomes (e) C-Mitosis and (f) Fragmented chromosomes (Scale bars = 200 μm).

In this research, various aberrations were visualized under the microscope such as stickiness, laggard, fragmented chromosomes and C-mitosis with variable occurrence for different mung bean varieties caused by Cd treatment in a concentration-dependent manner and negligible aberrations were observed in non-Cd-treated samples (Fig 2).

**Fig 3:**
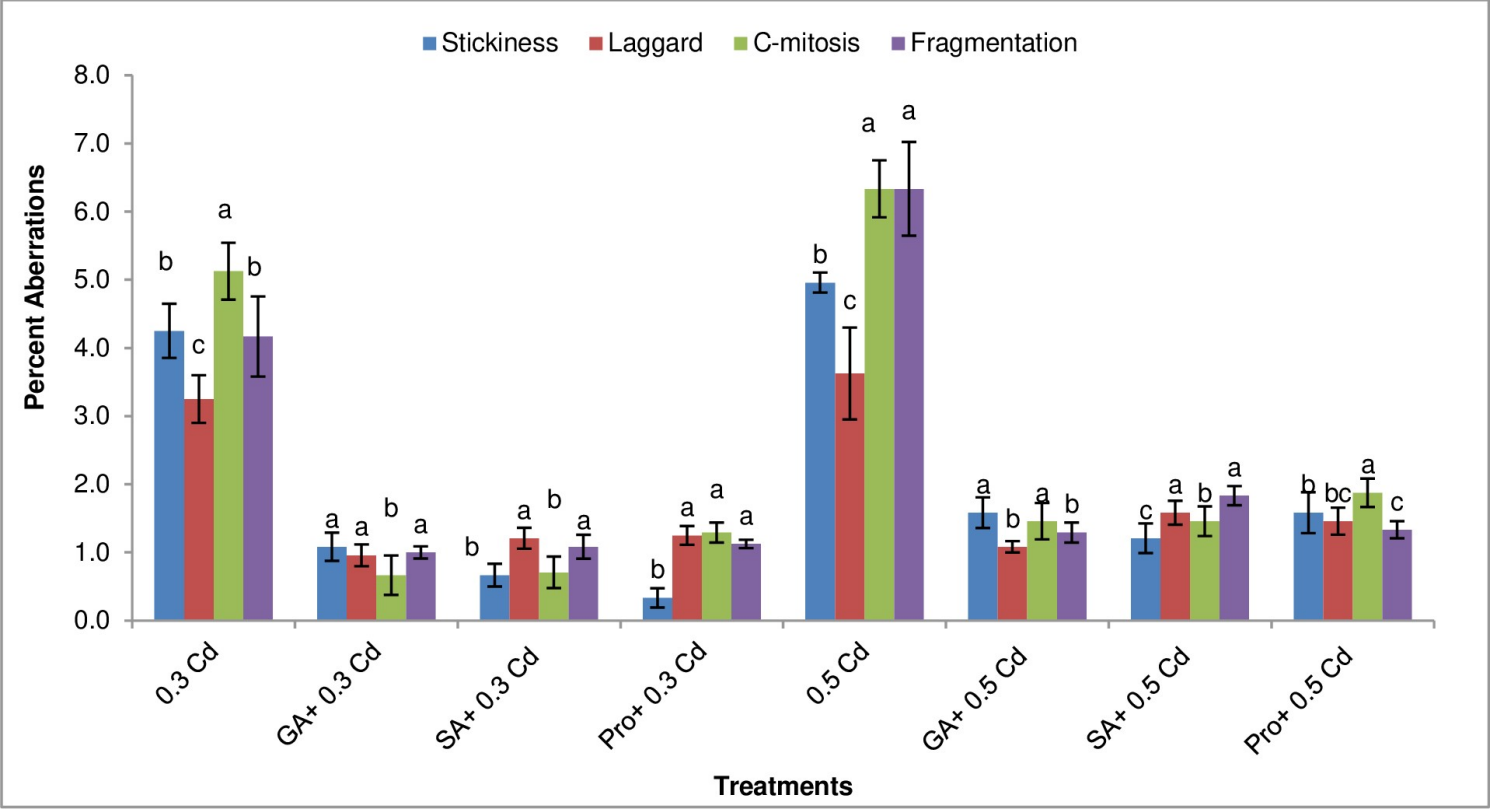
Percent chromosomal aberrations from mung bean root tips under Cd treatments alone and priming with phytohormones and proline. Different alphabets in graphs exhibits significant differences among the treatments. Vertical bars shows a standard error with n = 24 at the P ≤ 0.05 level of significance.

The higher value for C-mitosis (5%) was recorded at 0.3 mM Cd irrespective of variety, followed by stickiness (4%), fragmented (4%) and laggard (3%) chromosomes. Moreover, it was also observed that pretreatment of phytohormones and proline tends to decrease percent aberrations for all varieties at both Cd concentrations (Fig 3).

**Fig 4:**
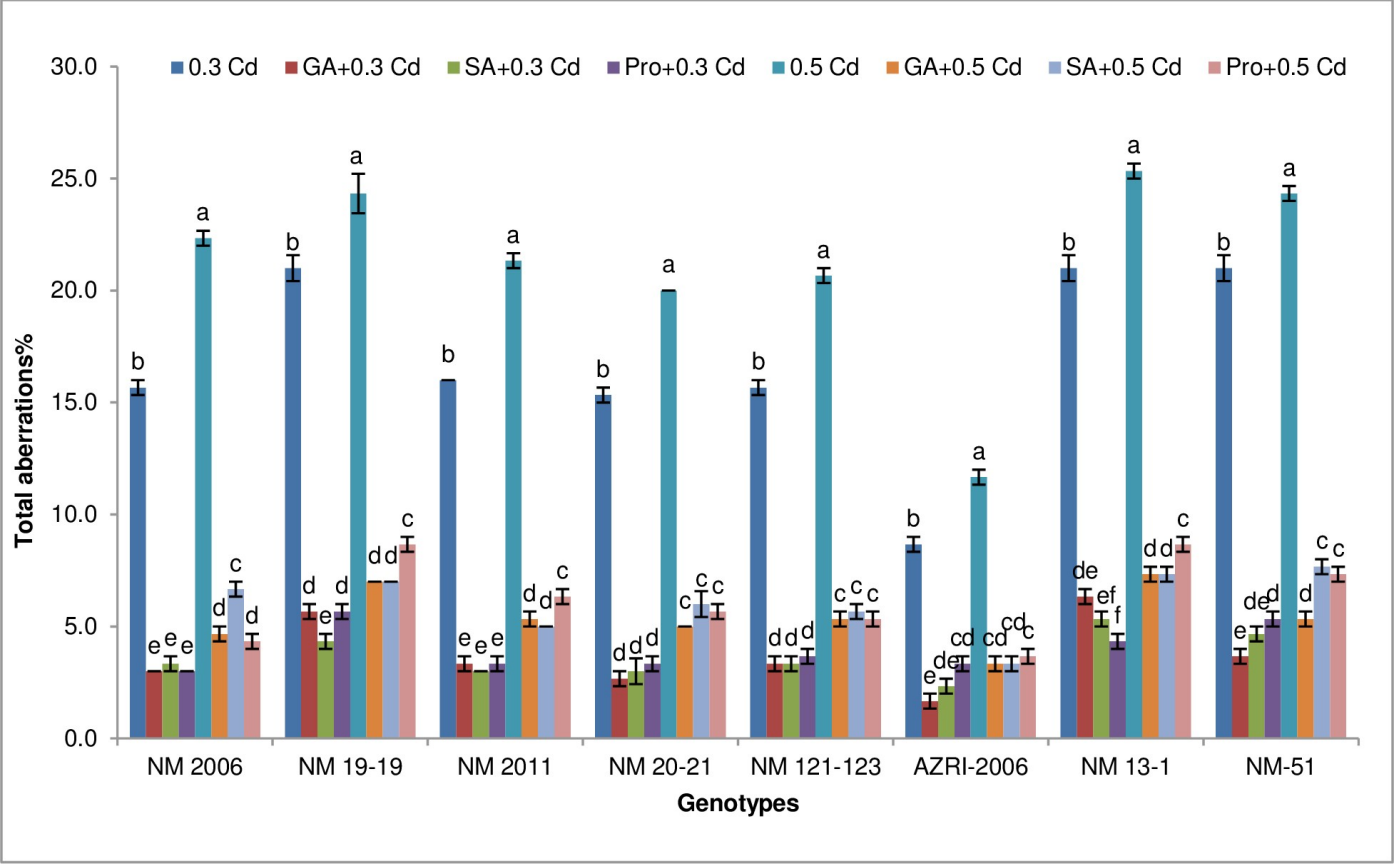
Total aberrations from mung bean root tips under Cd treatments alone and priming with phytohormones and proline prior to Cd stress. Different alphabets in graphs exhibits significant differences among the treatments for each variety. Vertical bars shows a standard error with n = 3 at the P ≤ 0.05 level of significance.

It was revealed that a higher frequency of chromosomal aberrations was found in NM 13–1, NM 19–19 and NM-51 at both Cd treatments (Fig 4). AZRI-2006 revealed the lowest frequency of aberrations 8% and 11% at 0.3 mM and 0.5 mM Cd respectively. However, when seeds were pre-treated with GA_3_, SA and proline before Cd stress tends to decrease these aberrations in cells of all varieties tested here (Fig 4).

### Effect of Cd on growth traits

After 1 week of Cd treatment seedling length, relative water content, fresh and dry weight was measured. Highly significant differences between genotypes and treatments along with interaction were observed for all growth parameters as indicated in Table 2.

**Table 2:** Mean sum of squares for morphological parameters of eight mung bean genotypes grown under Cd and pretreatments of GA_3_, SA and proline prior to Cd.

SOV	Df	MS			
		SL	FW	DW	RWC
Genotypes	7	50.592**	0.091**	0.0009**	34.08**
Treatments	8	211.562**	0.031**	0.00003**	9.94**
G×T	56	5.912**	0.003**	0.00001**	1.719**
Error	144	0.051	0.00004	0.0000013	0.294

Results demonstrated that Cd have adverse effects on plant growth as indicated by reduction in seedling length, fresh and dry weight on a concentration-dependent manner. However, the pretreatment with phytohormones (GA_3_ and SA) and proline prior to Cd alleviated the adverse effects of Cd represented by improvement in these traits (indicated in S2 Fig)

### Seedling length

**Fig 5:**
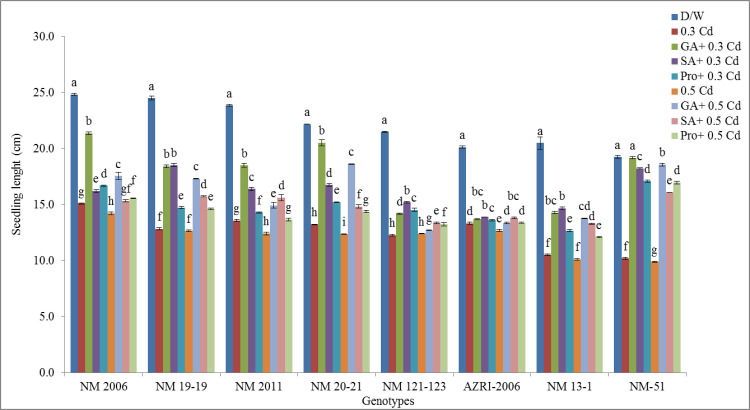
Mean seedling length of mung bean genotypes grown under Cd and pretreatments of GA_3_, SA and proline prior to Cd.

The highest inhibition in seedling length was observed in NM 13–1 (48.76%) followed by NM 19–19 (47.62%) and NM-51(47.09%) at 0.3 mM Cd. Similarly, at 0.5 mM Cd, inhibition in seedling length was highest in NM 13–1 (50.63%), then NM-51 (48.61%) and NM 19–19 (48.37%). Under 0.3 mM Cd and 0.5 mM Cd AZRI-2006 shows the least reduction in seedling length i.e. 33.76% and 36.99% respectively. The lowest inhibition was observed in NM-51 with pre-treatment of GA_3_ (0.43% and 3.60%), SA (5.36% and 16.50%) and proline (11.21% and 11.93%) at 0.3 and 0.5 mM Cd respectively (Fig 5).

### Fresh weight

**Fig 6:**
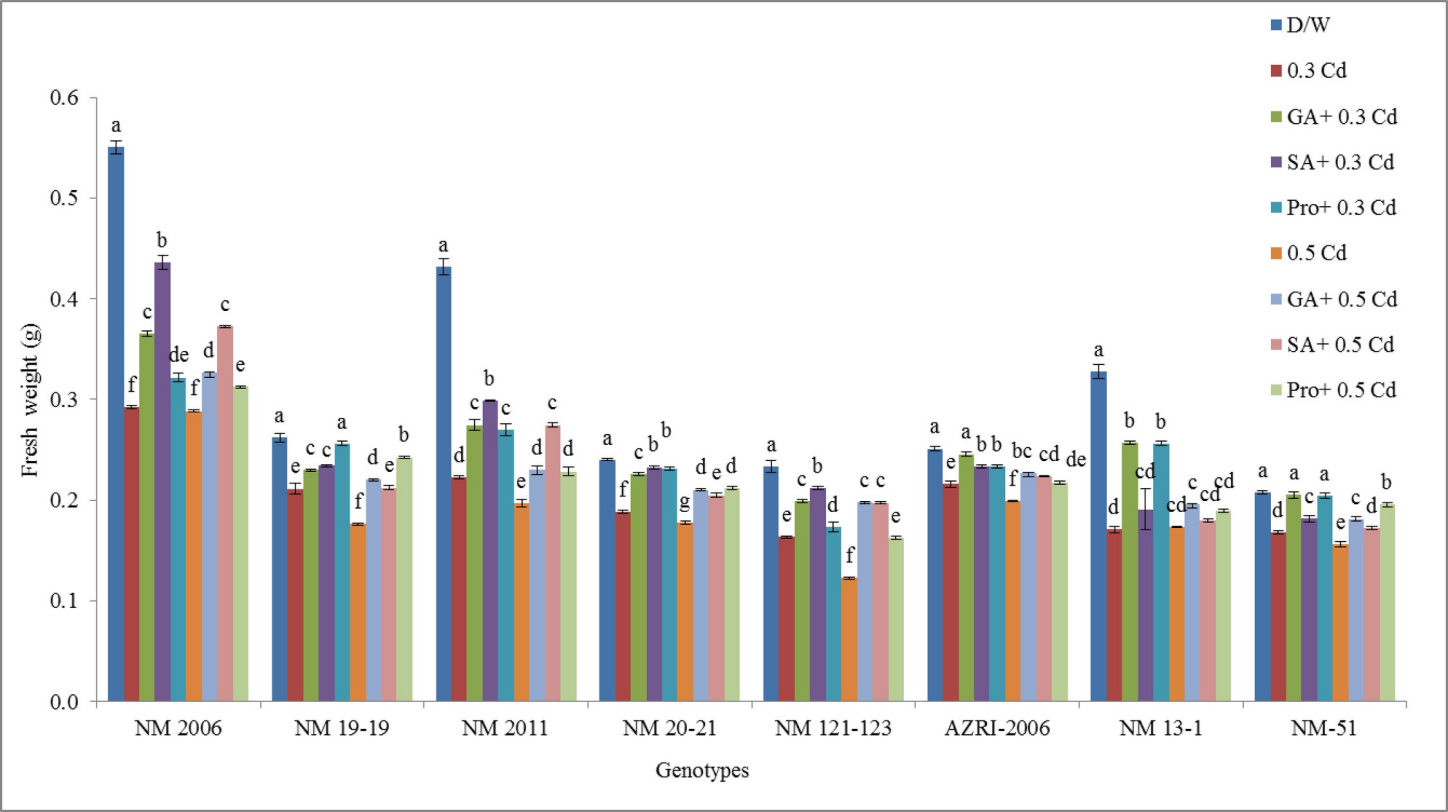
Mean fresh weight of mung bean grown under Cd and pretreatments of GA_3_, SA and proline prior to Cd.

The highest inhibition in fresh weight was observed in NM 2011 at 0.5 mM Cd with 54.44% inhibition while the lowest inhibition in fresh weight was observed in AZRI-2006 (13.94%) at 0.3 mM Cd as compared to control as shown in S3 Fig. Results also demonstrated that phytohormones and proline were helpful in partially alleviating the negative effects of Cd on mung bean seedlings. Alleviation was more prominent after priming with phytohormones and proline in NM 20–21, AZRI-2006 and NM-51 genotypes as compared to control. It was also worth mentioning that different genotypes have different interactions with both phytohormones and proline during Cd stress (Fig 6).

### Dry weight

**Fig 7:**
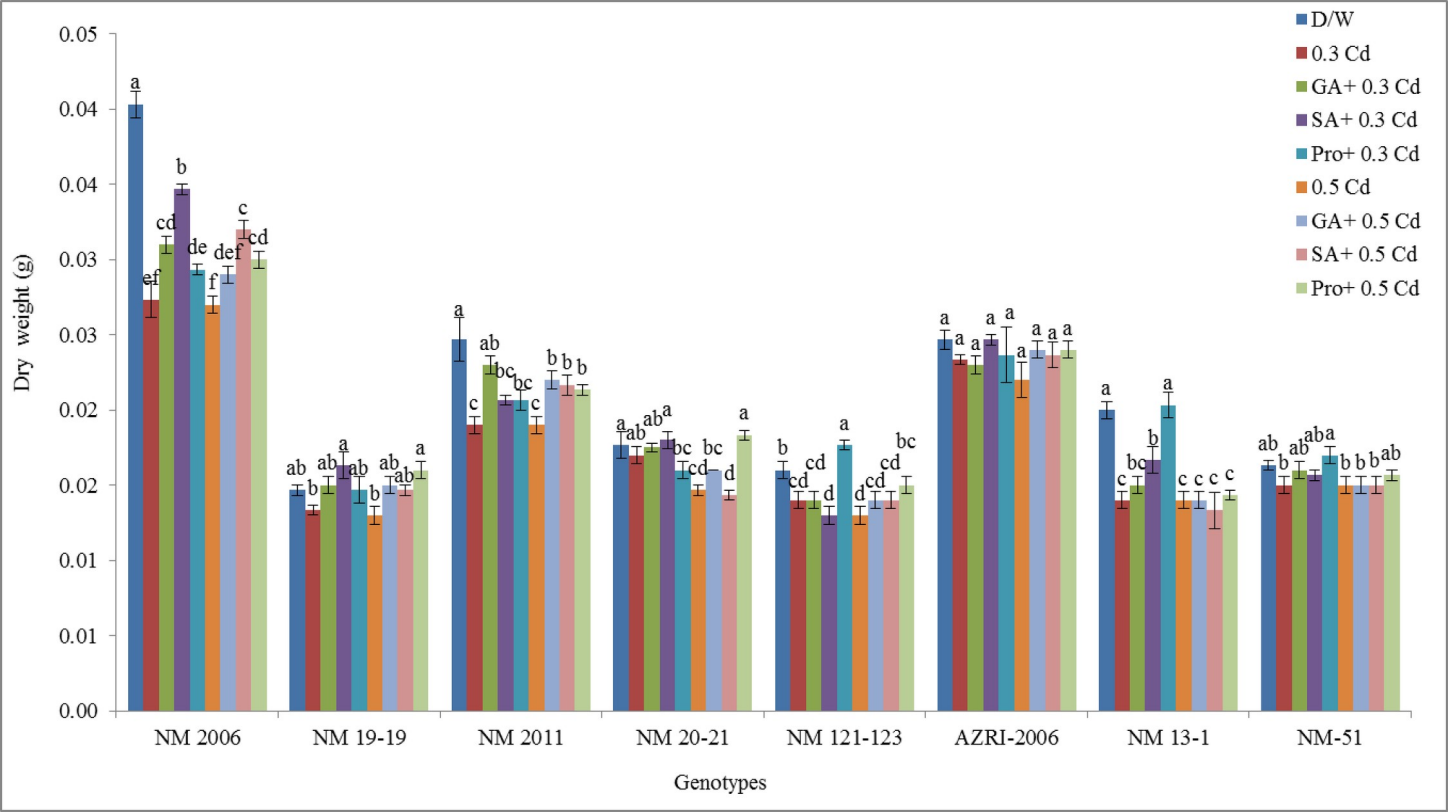
Mean dry weight of mung bean grown under Cd and pretreatments of GA_3_, SA and proline prior to Cd.

Results indicated that least inhibition in dry weight was observed in NM 20–21 (3.74%) and NM-51 (8.16%) at 0.3 mM Cd (shown in S4 Fig). Surprisingly in genotype AZRI-2006 non-significant difference in dry weight was observed in all treatments with respect to control even SA tends to keep dry weight unchanged (Fig 7).

The highest inhibition in dry weight was found in NM 2006 (32.20% and 33.10%) at 0.3 mM and 0.5 mM Cd respectively. NM 19–19, NM-51 and AZRI-2006 were least affected by Cd treatment with respect to dry weight. It was further noticed that there was a promotion in dry weight for all priming treatments in genotype NM 19–19, NM 2011 and NM-51 as compared to metal alone. The least alleviation in Cd toxicity was observed in NM 13–1 and NM 2006 under all pretreatments. There was variable response of different pretreatments on genotypes.

### Relative water content

**Fig 8:**
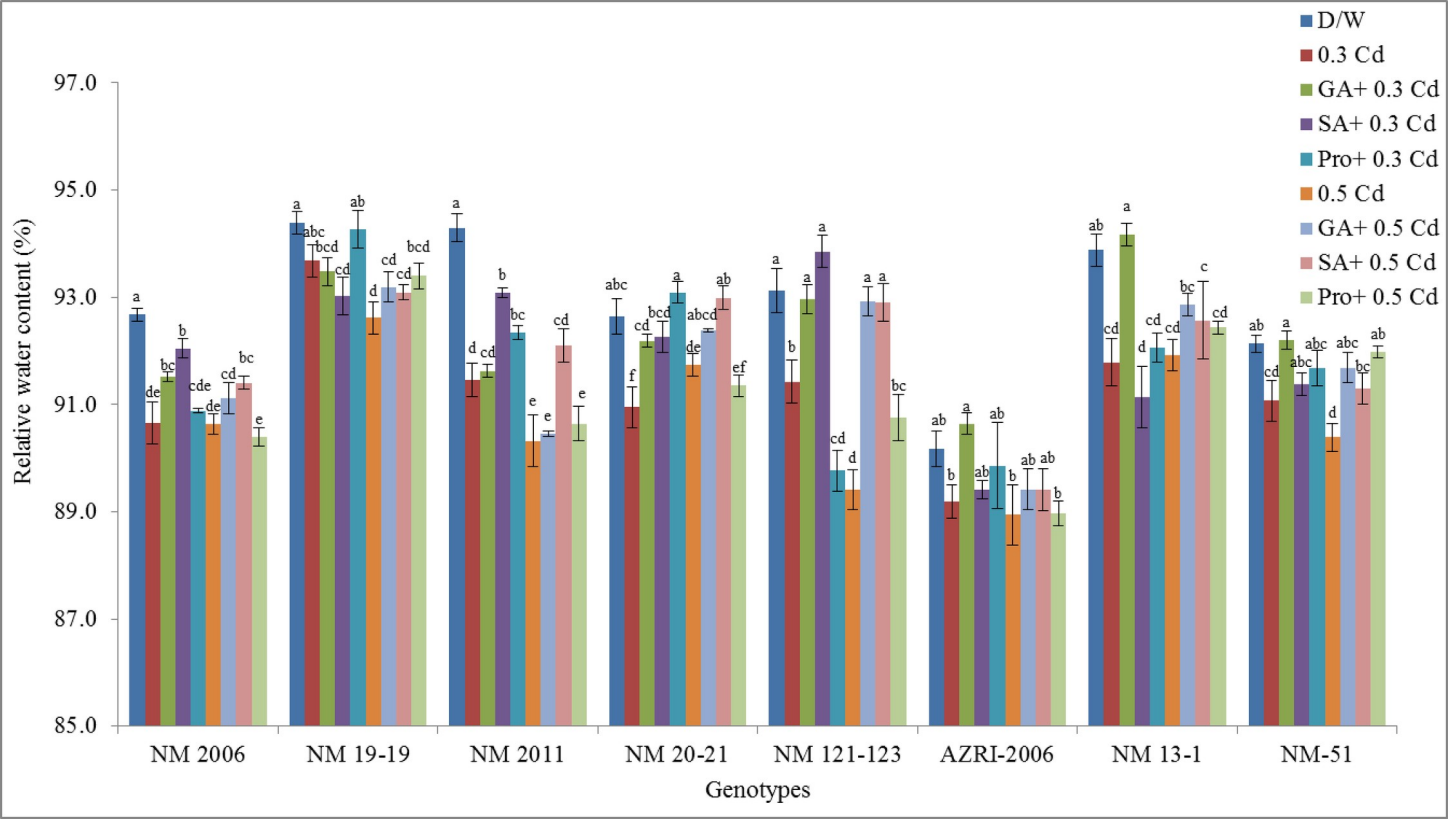
Mean relative water content of mung bean grown under Cd and pretreatments of GA_3_, SA and proline prior to Cd.

There was a significant decrease in mean relative water content in Cd treated seedlings with respect to control in a concentration-dependent manner (Fig 8).

While it was clearly observed that pretreatment of phytohormones and proline helped in reducing Cd toxicity on mung bean seedlings, with a variable genotypic response. In NM 20–21 under SA and GA_3_ pretreatment at 0.3 mM Cd increased in RWC was observed as compared to 0.3 mM Cd alone, however pretreatment with proline, was even higher than in control. The same results were obtained for NM 121–123 under SA pretreatment at 0.3 mM Cd. GA_3_ at 0.3 mM Cd also tends to increase RWC in NM 13–1 and NM-51. Results showed that relative water contents were severely affected in genotype NM 2011 (2.71% and 5.09%) in both the Cd treatments respectively. Whereas least inhibition in RWC was observed in NM 19–19 at 0.3 mM Cd (0.76%) treatment and in NM 20–21 (0.97%) at 0.5 mM Cd (shown in S5 Fig). Variable response of genotypes under pretreatments was also detected. RWC was found higher than control of few genotypes under some pretreatment at stress.

### Alteration in antioxidant enzymes (APX, CAT, GPX, SOD) under stress

Four different antioxidant enzymes activities were analyzed in mung bean seedlings under Cd treatment along priming with phytohormones and proline prior to Cd. Table 3 shows that in all enzymes activities, genotypes and treatments were significantly different. Similarly, the interaction between genotypes and treatments was found to be highly significant (Table 3).

**Table 3:** Mean sum of squares for antioxidant enzymes, protein, MDA and proline of eight mung bean genotypes grown under Cd and pretreatments of GA_3_, SA and proline prior to Cd for 1 week.

SOV	Df	MS						
		APX	CAT	GPX	SOD	Protein	MDA	Proline
Genotypes	7	29.778**	0.186**	10675.649**	968.489**	251688.449**	4052.844**	24376.923**
Treatments	8	12.496**	0.095**	1322.377**	133.224**	76791.241**	8364.961**	6955.175**
G×T	56	2.678**	0.013**	334.351**	34.928**	5077.622**	276.735**	473.849**
Error	144	0.003	0.00002	0.376	0.059	11.718	0.246	0.238

### Ascorbate peroxidase

**Fig 9:**
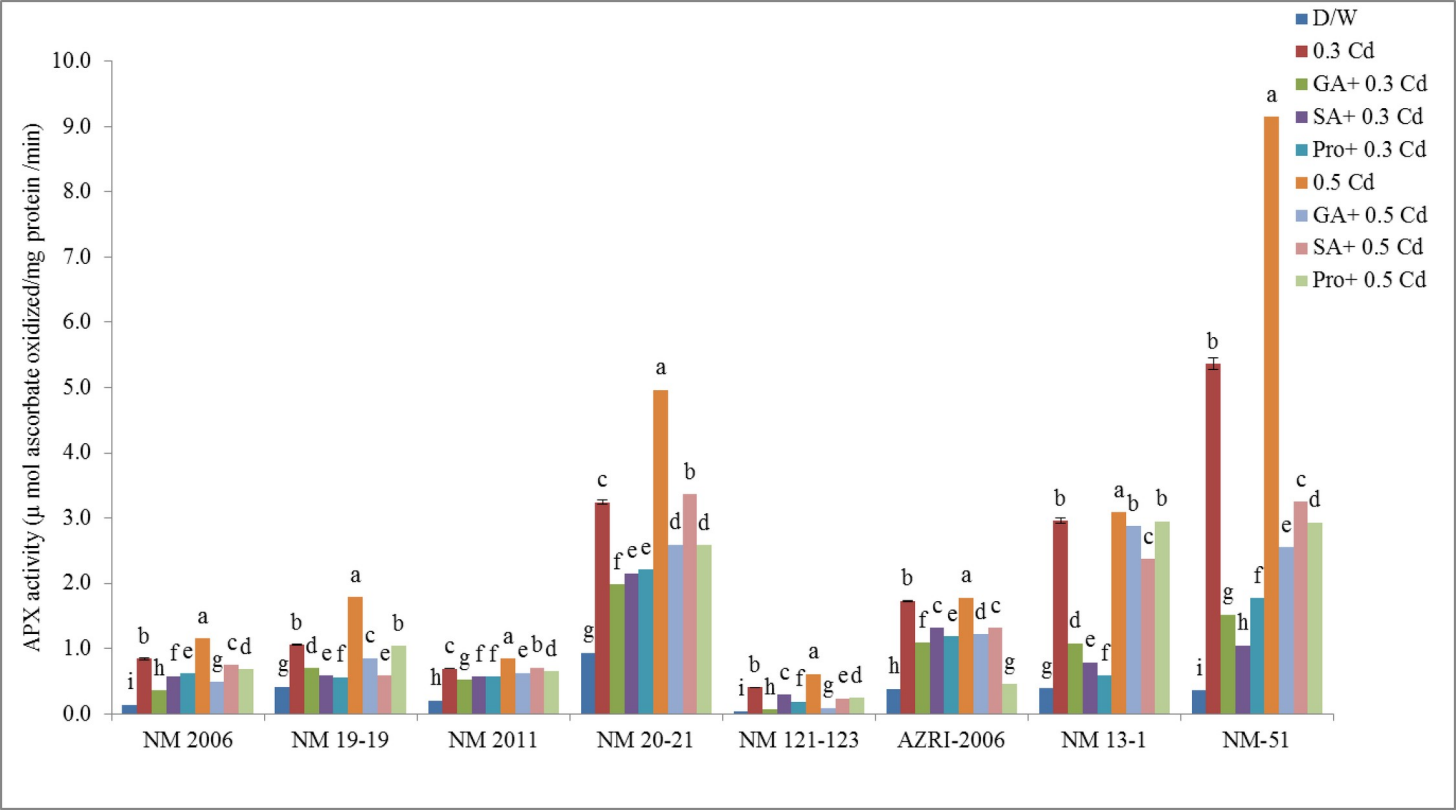
Specific activity of APX enzyme mung bean grown under Cd and pretreatments of GA_3_, SA and proline prior to Cd.

Results revealed that Cd treatment was responsible for the activation of APX enzymes in mung bean seedlings. The induced activity of the APX enzyme was noted in Cd-treated seedlings in a concentration-dependent manner. NM-51 showed the highest activities at both the Cd concentrations while lower activity was observed in NM 121–123 among all genotypes as illustrated in Fig 9.

It was also noticed that pretreatment with GA_3_, SA and Proline was able to cause a decrease in Cd toxicity by decreasing APX enzyme as compared to Cd but was still higher than in control. The highest promotion in APX activity was observed in NM-51 at 0.5 mM Cd. While NM 19–19 showed the least promotion in APX activities under all pretreatments (shown in S6 Fig).

### Catalase

**Fig 10:**
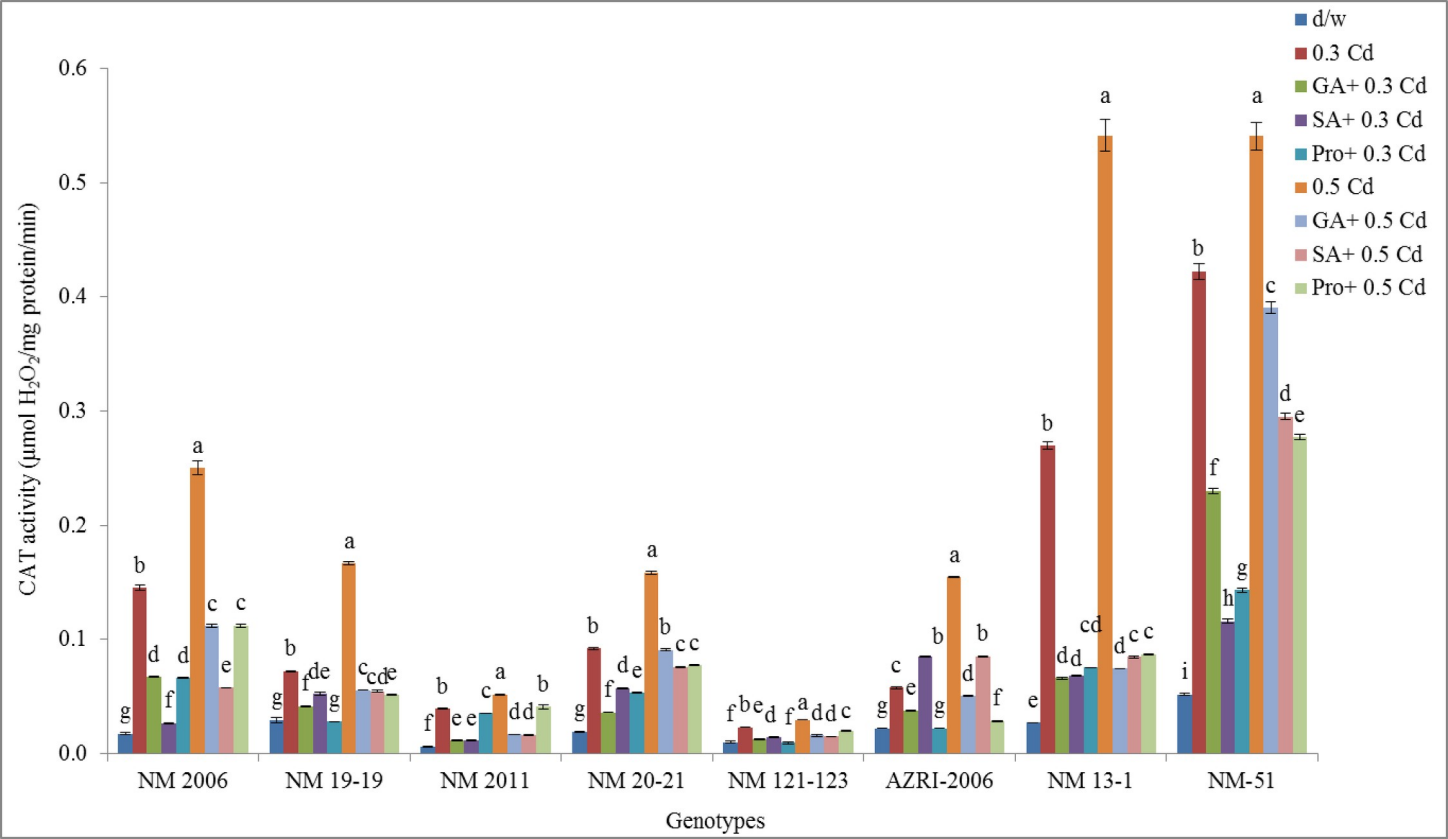
Catalase activity of mung bean grown under Cd and pretreatments of GA_3_, SA and proline prior to Cd.

It was demonstrated by the graph that CAT was increased by increasing Cd concentration. Among all genotypes, NM-51 and NM 13–1 have higher values of catalase at both the Cd treatments. While lower activities were observed in NM 19–19, NM 121–123 and AZRI-2006. While pretreatment with GA_3_, SA and proline prior to Cd tends to cause a decrease in CAT activities in all genotypes however these values were still higher than in control (Fig 10).

While higher promotion in CAT activity was observed in NM-51, NM 13–1 and NM 2006 at both the Cd concentrations. The lowest activity for catalase were noticed in NM 121–123. While pretreatment with phytohormones before Cd tends to decrease catalase activity but is still higher than control. In Azri-2006 proline before 0.5 mM Cd was able to maintain this enzyme activity near to control (indicated in S7 Fig).

### Guaiacol peroxidase

**Fig 11:**
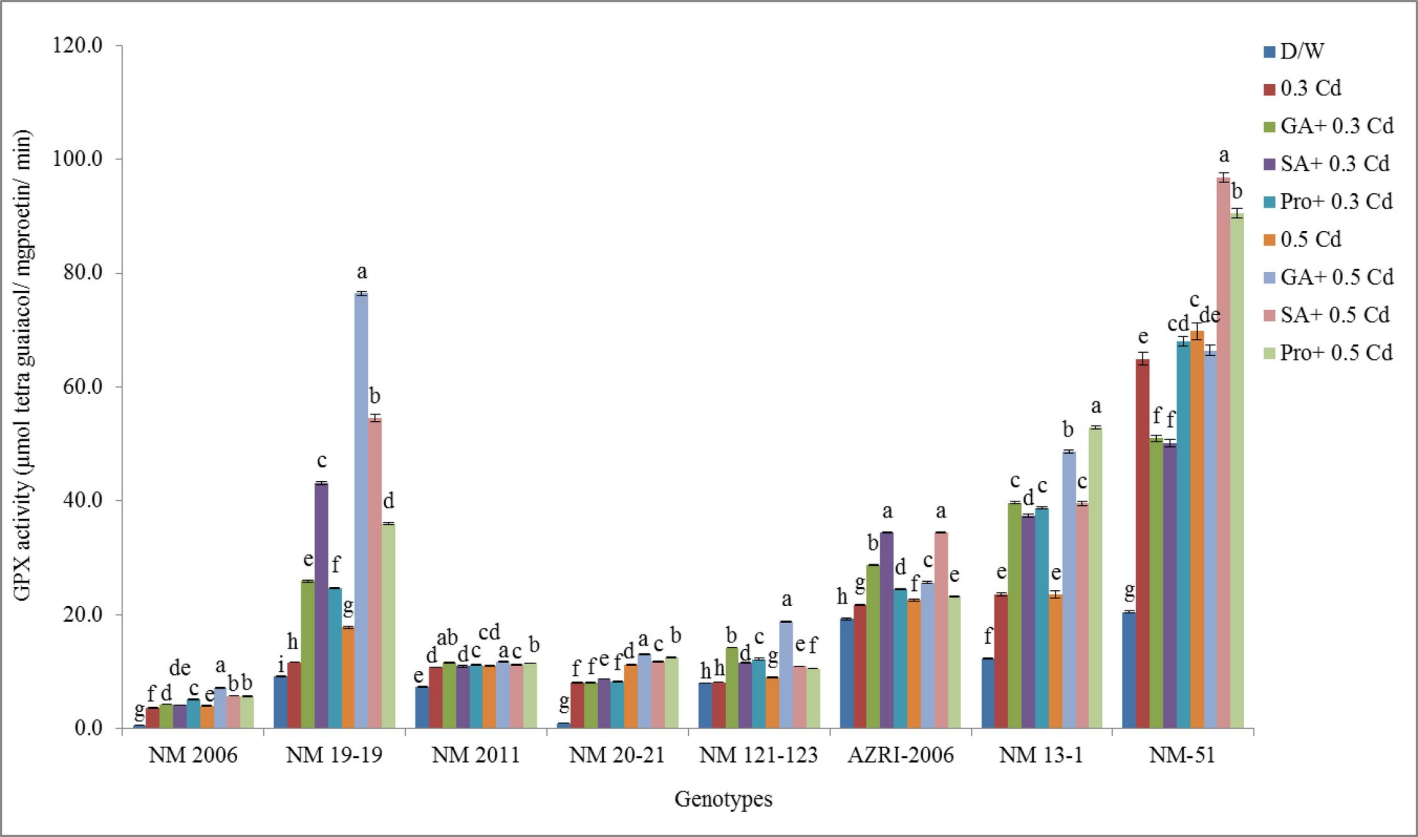
Mean values for GPX activity in mung bean grown under Cd and pretreatments of GA_3_, SA and proline prior to Cd.

Cd treatment was responsible for increasing GPX activity in a concentration-dependent manner in most of the genotypes. NM 2006 shows the least values for GPX enzymes while higher values were observed in NM-51 under all treatments as well as in control (Fig 11).

While all the phytohormones before Cd tend to further promote the GPX activities in most of the genotypes with the non-significant promotion in few genotypes. While highest promotion in GPX activity were observed in pretreated samples of NM 20–21 and NM 2006 as compared to control (indicated in S8 Fig).

### Superoxide dismutase

**Fig 12:**
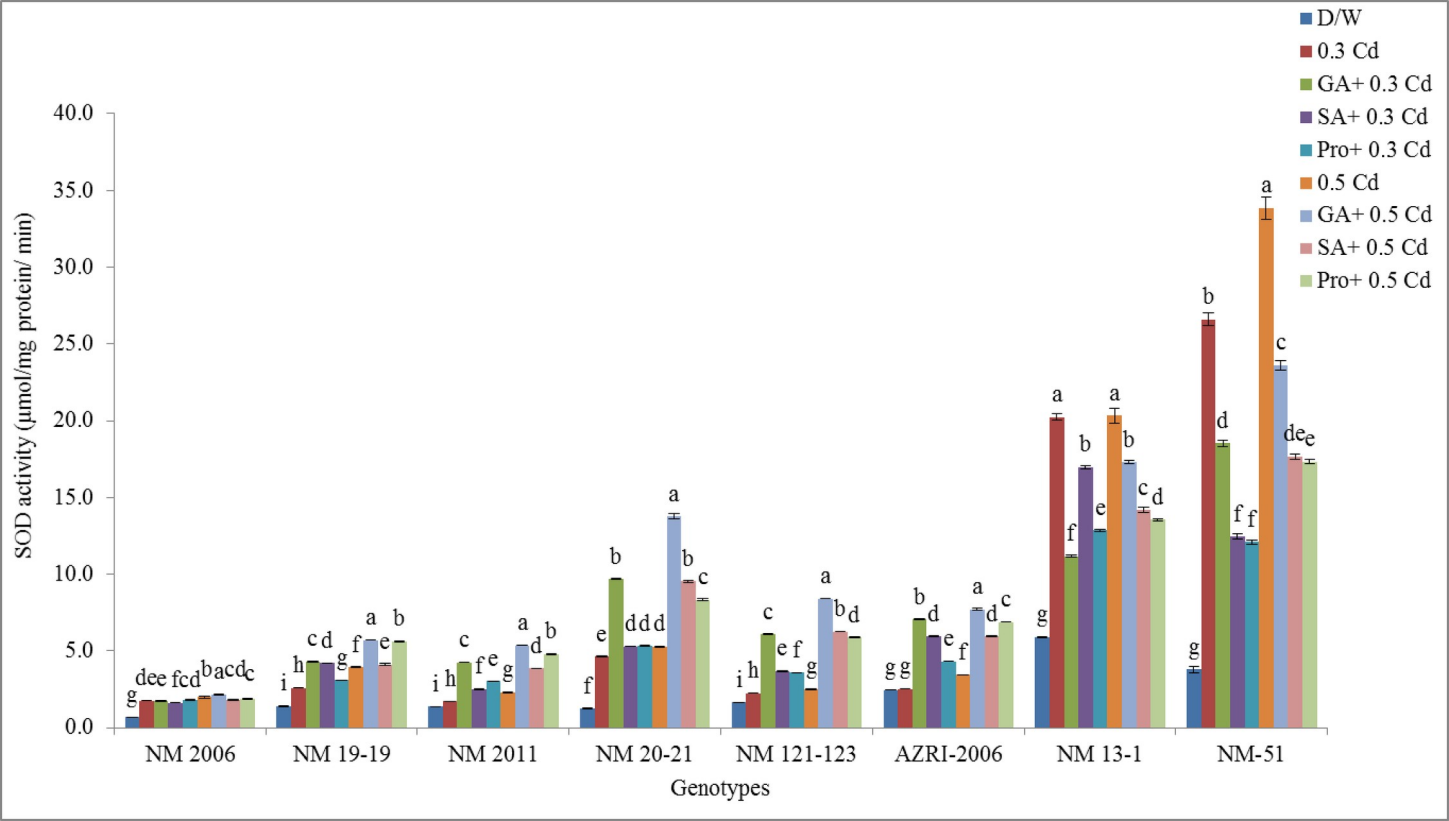
Mean SOD activities of mung bean grown under Cd and pretreatments of GA_3_, SA and proline prior to Cd.

SOD is an important antioxidative enzyme. SOD activities showed different patterns among eight mung bean genotypes. The lowest activity under control as well as all treatments was measured in NM 2006 while NM 13–1 and NM-51 showed higher values for SOD. It was detected that SOD activity was increased under Cd treatment of all genotypes as compared to control. Pretreatments with phytohormones and proline before Cd showed a decrease in SOD activity of NM 13–1, NM-51 and NM 2006 as compared to metal stress alone. While other genotypes exhibited an increase in SOD activity when pretreated with phytohormones and proline as compared to Cd stress (Fig 12).

The lowest promotion in SOD activity was observed in AZRI-2006 for all the treatments. While highest promotion values were observed in NM 20–21 under all the pretreatments (shown in S9 Fig).

### Effect of Cd stress on biochemical (Protein, MDA, Proline) attributes

In 1 week Cd exposed mung bean seedlings recorded biochemical parameters were protein, proline, and MDA contents.

Statistical analysis for biochemical parameters showed that all the estimated parameters like protein, MDA, and proline were highly significantly different for genotypes and treatment. Table 3 also demonstrated a highly significant interaction between both factors.

### Total protein contents

**Fig 13:**
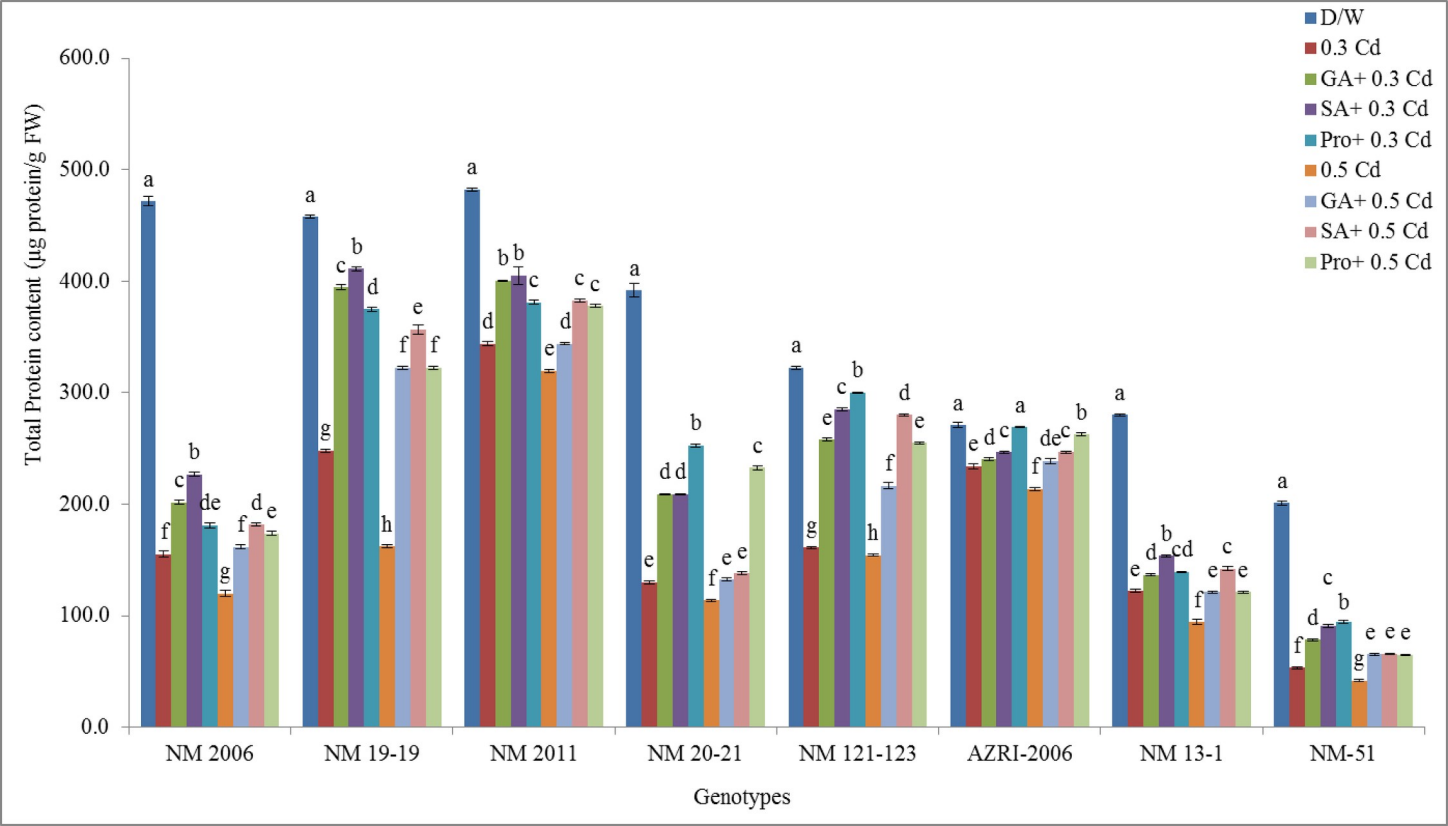
Mean total protein contents of mung bean grown under Cd and pretreatments of GA_3_, SA and proline prior to Cd.

Total soluble proteins were quantified on UV-Spectrophotometer at 750 nm. It was noticed that the highest protein values were observed in NM 2011 (482.33 μg/g FW) while lowest in NM-51 (201 μg/g FW) under control conditions. While Cd treatment gradually decreases protein contents in a concentration-dependent manner for all genotypes. The lowest value for protein was observed in NM-51 (41.66 μg/g FW) at 0.5 mM Cd treatment. All pretreatments specifically SA and proline were found to be more potent that tend to alleviate the Cd toxicity by increasing protein contents as compared to Cd alone but were still lower than control (Fig 13).

The lowest reduction in protein content as compared to control was observed in AZRI-2006 under 0.3 mM and 0.5 mM Cd treatments (13.53% & 21.16% respectively) as well as under all the pretreatments. Whilst highest percent inhibition was observed in NM-51 at 0.3 and 0.5 mM Cd (73.47% and 79.27% respectively) (shown in S10 Fig).

### Lipid peroxidation (MDA contents)

**Fig 14:**
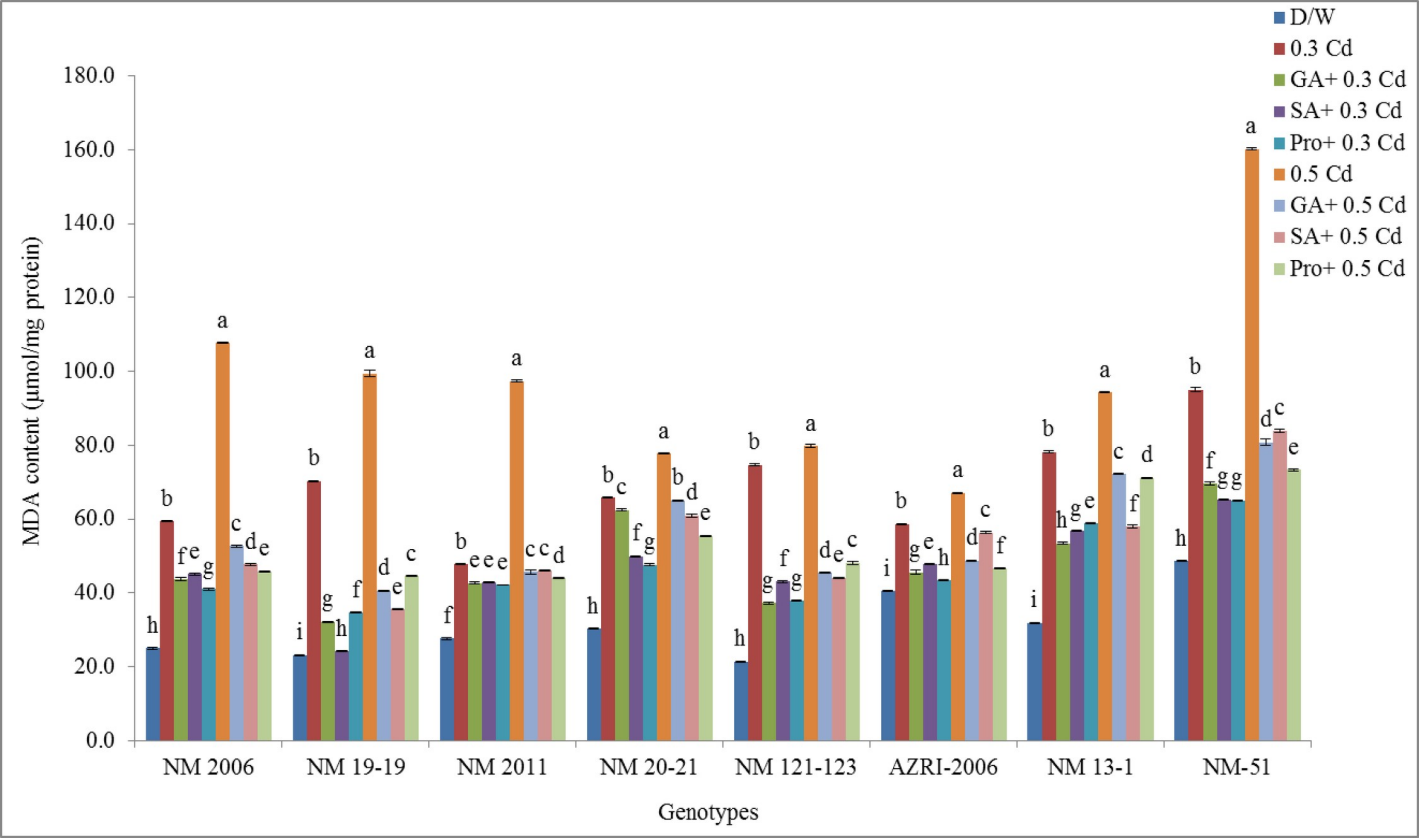
MDA contents of mung bean grown under Cd and pretreatments of GA_3_, SA and proline prior to Cd.

It was observed that Cd tends to cause lipid peroxidation in mung bean seedlings that were calculated as MDA content. The highest values for MDA content were found to be in NM-51 at all treatments as well as at 0.3 and 0.5 mM Cd (95.06 and 160.24 μmol/μg protein) respectively. It was indicated from results that pretreatment helped in decreasing lipid peroxidation and membrane damage as indicated by a decrease in MDA values for all genotypes. The lowest values for MDA content were observed in NM 19–19 under all pretreatments at both the Cd concentrations as shown in Fig 14.

It was further noticed that proline pretreatment prior to Cd supply was more effective in lowering MDA contents at both the Cd concentrations in most genotypes. NM 121–123 showed the highest promotion (248.48%) while AZRI-2006 showed the lowest (44.80%) at 0.3 mM Cd among all genotypes. More improvement in membrane damage was observed in AZRI-2006 by phytohormones at both the Cd concentrations indicated by lowered MDA content. At a higher concentration of Cd, both NM 2006 and NM 19–19 showed approx. similar promotion values for MDA (329.81 & 329.80%) respectively however least values were found in AZRI-2006 (65.50%) (shown in S11 Fig). The above results showed that Cd was responsible for high membrane damage while pretreatments of phytohormones and proline prior to Cd reduced the membrane damage and protect the cells.

### Proline contents

**Fig 15:**
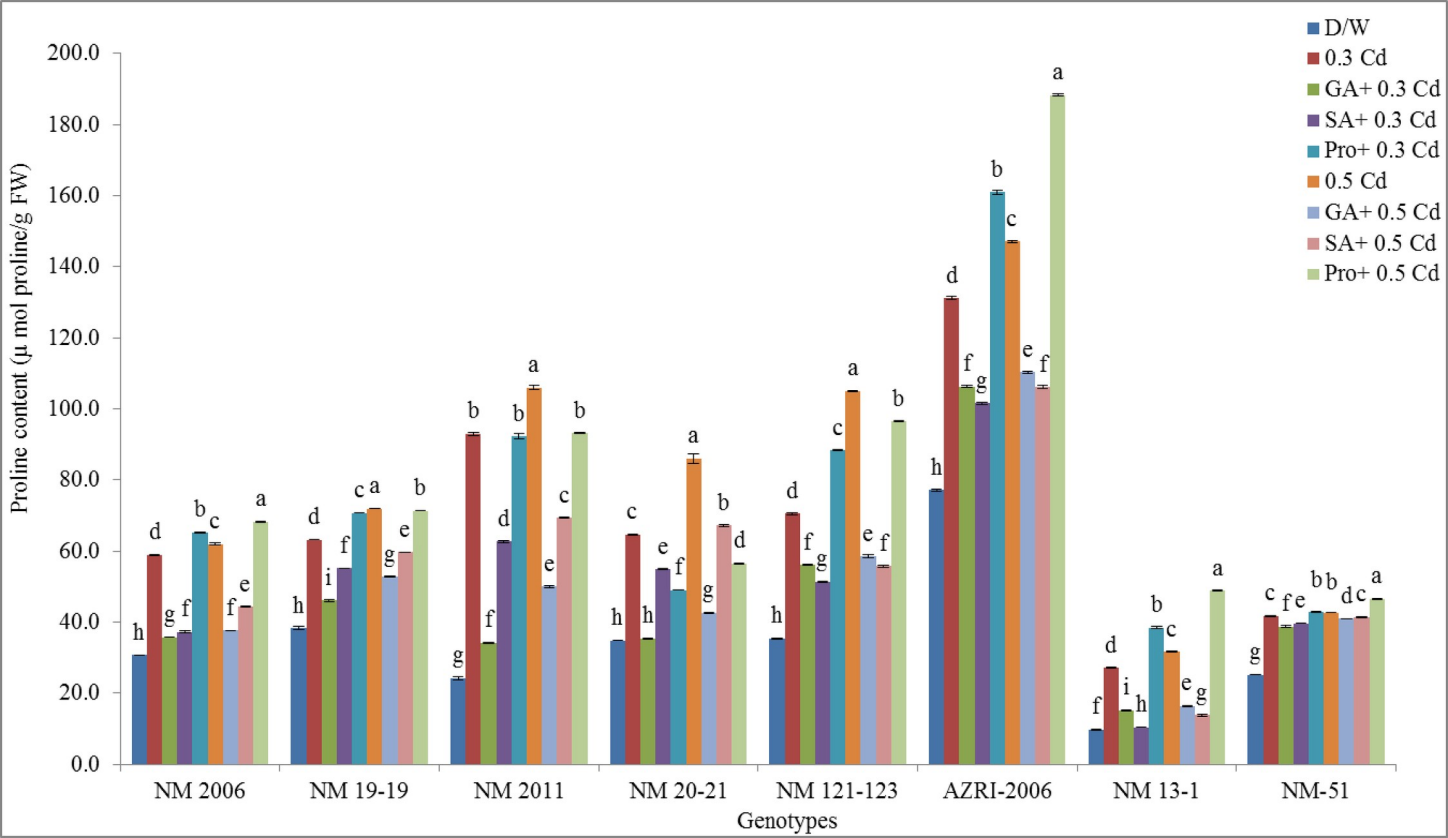
Mean proline contents of mung bean grown under Cd and pretreatments of GA_3_, SA and proline prior to Cd.

Increased Cd concentration tends to elevate the proline contents in mung bean seedlings as compared to control. Under control treatment, genotypes behave in the same way by producing the lowest proline as compared to any treatment. AZRI-2006 showed the highest proline contents (77.18 μmol/g FW) while NM 13–1 showed the least values (9.74 μmol/g FW) in control among all genotypes. It was noticed that the pretreatments before Cd were able to reduce proline content. However, promotion in proline was seen in all genotypes except NM 20–21 when seeds were imbibed in proline and then treated with Cd (Fig 15).

Proline accumulation under both the Cd treatment was in a dose-dependent manner in all mung bean seedlings. The highest promotion in proline contents was noticed in NM 2011 (284.90% and 338.00% at 0.3 & 0.5 mM Cd respectively) followed by NM 13–1. However, NM 19–19, AZRI-2006 and NM-51 showed a similar pattern for proline percent promotion in all treatments. Surprisingly it was further noticed that in genotype NM 13–1 percent promotion in proline pretreated seedlings was higher among all genotypes (295.10% and 401.80% at 0.3 and 0.5 mM Cd after proline pretreatment respectively) (shown in S12 Fig).

Pearson correlation among different parameters had been tested during this experiment. Morphological parameters (except with DW with RWC), protein, mitotic index and activities were found to be positively correlated among each other. While MDA, antioxidant enzymes and total aberration% were found to be negatively correlated with all growth parameters and protein. Proline negatively correlated with antioxidant enzymes but was non-significant with MDA, while all the antioxidant enzymes and MDA contents were found to have a significant positive correlation as indicated in Table 4.

**Table 4:** Pearson correlation coefficient among all tested parameters in mung bean seedlings grown under Cd stress with prior application of phytohormones and proline.

	FW	DW	RWC	Proline	MDA	Protein	APX	CAT	GPX	SOD	MI	Aberra
SL	0.56**	0.30**	0.37**	-0.38**	-0.56**	0.44**	-0.40**	-0.32**	-0.15*	-0.32**	0.61**	-0.65**
FW		0.82**	0.20**	-0.20**	-0.46**	.044**	-0.40**	-0.31**	-0.40**	-0.46**	0.60**	-0.42**
DW			-0.38**	0.16*	-0.27**	0.22**	-0.31**	-0.20**	-0.38**	-0.42**	0.60**	-0.29**
RWC				-0.61**	-0.30**	0.27**	-0.13	-0.16*	0.004	-0.03	-0.04	-0.18**
Proline					-0.01	0.16*	-0.11	-0.19**	-0.15*	-0.32**	0.22**	0.09
MDA						-0.72**	0.76**	0.77**	0.37**	0.64**	-0.75**	0.79**
Protein							-0.60**	-0.65**	-0.43**	-0.65**	0.56**	-0.44**
APX								0.75**	0.46**	0.74**	-0.61**	0.52**
CAT									0.53**	0.77**	-0.68**	0.58**
GPX										0.70**	-0.40**	0.09
SOD											-0.70**	0.30**
MI												-0.74**

## Discussion

In developing countries, good quality fertilizers are expensive to be used by the poor farmers so interchangeably they use sewage/industrial wastewater and cheap fertilizers to irrigate their crops which contain a large amount of various heavy metals. These heavy metals may absorb and reside in plants and can cause toxic effects to human beings or farm animals when consumed. On the other hand, heavy metals like Cd exhibited negative effects on the growth of plants which ultimately reduce the yield of the crop. This study was performed to analyze the clastogenic effect of Cd on mung bean seedlings and to find out whether phytohormones and proline help in acclimatizing cytogenetic effects and oxidative stress due to Cd. This study showed that Cd tends to decrease mitotic division and cause different chromosomal disorders in all mung bean varieties. AZRI-2006 showed better values for cell division (MI%) under Cd concentrations as compared to other varieties as indicated in the results section that showed its better genetic potential against Cd. Whereas, NM-51 was found to be the least resistant to Cd at both concentrations. It has been studied that binding of Cd with sulfhydryl group (-SH) of enzymes inhibits mitotic index [33] and decline in MI suggested that the Cd could prevent cells from going into cell division. Similar results regarding mitotic index under cadmium stress were also reported in other crops including *Vigna radiata* [8, 34], lentils [35], cowpea [36], broad bean [37, 38].

The decrease in MI in Cd-treated root was might be due to disturbances during the process of chromatin formation in the cell cycle that resulted due to interactions between DNA and heavy metals [39]. Reduction in root growth and cell division under cadmium stress was prominent in other plants. It has been described that Cd tends to disturb roots structure by damaging cell nucleoli and inhibits the repair mechanics of DNA [40].

Results of the present work demonstrated various chromosomal abnormalities included C-mitosis, sticky chromosomes, laggards and fragmented that were due to distractions in spindle fibers during cell division of mung bean. Oxidative stress caused by Cd in cowpea is the main cause of damaged DNA that leads to multiple anomalies in chromosomes during cell division, like strand breaks, removal and alterations in nitrogenous basis [36]. Fragments observed in mitotic slides under Cd stress were formed due to the stickiness of chromosomes and chromatids that were not separated at the poles. Cd-treated samples exhibited fragmented chromosomes in all varieties. It was also suggested that ROS tends to cause breakage in a double strand of DNA [41]. The presence of laggard chromosomes in treated samples was formed by instabilities in the spindle apparatus or the centromere. These results are in accordance with [42] who reported that weak C-mitotic effect was responsible for chromosome lagging which may lead to aneuploidy.

There are certain strategies to reduce environmental stresses like heavy metal toxicity in plants, among them an appropriate dose of phytohormones is also a major approach [43]. In this research, we have done priming of seeds with phytohormones (GA_3_ and SA) and proline prior to Cd stress. Researchers reported that under control conditions, GA_3_ acts as initiating source to activate loading and transportation of mineral elements within the plants [44]. In many plants, heavy metals may alter the endogenous quantity of phytohormones so an exogenous application of phytohormones helps in reducing metal uptake from the soil that ultimately declines metal toxicity [45], as shown in exogenously supplied GA_3_ under Cd stress stimulates growth in tomato plants [46]. Interestingly in proposed research, mung bean seeds pretreated with GA_3_ tend to increase mitotic index grown under Cd stress as compared with non-pretreated seedlings and cause a reduction in chromosomal aberrations. So, it can be deduced that priming with GA_3_ tends to mitigate the adverse effects of Cd on mung bean cell division. Similarly, other scientists also reported the potentiality of GA_3_ in alleviating the detrimental effect of metals hence proves its beneficial role in acclimatizing metal stress on Arabidopsis and Lupin [47]. Furthermore, GA_3_ treatment greatly increased protein as compared to Cd treated plants as reported earlier [48].

Previous research showed the protective role of SA on various crops in signaling under metal toxicity [49]. It has also been suggested that SA facilitates normal growth and has the potential to alleviate Cd toxicity by phytoremediation [50]. Cd tends to decrease mitotic index and increase chromosomal abnormalities in rice, but SA priming of seeds showed an increase in MI% and alleviates Cd adverse effects to promote better growth. Furthermore, it is reported that SA has the potential to increase agriculture production in terms of quality and quantity under various abiotic stresses including metals [51].

Our results showed a decrease in seedling length and biomass with increasing Cd concentration are in accordance with other studies done on mung bean or other crops [52–55]. In our results, AZRI-2006 showed better growth under Cd treatments that can be attributed to its better genetic potential as has been observed in other mung bean cultivars [56]. In our results, the Cd stress to mung bean seedlings seemed to reduce the availability of water to plant, and this may be the reason for the reduction of seedling growth in our experiment supported by other work [57, 58]. In this research, decrease in soluble protein was also recorded in Cd treated seedlings in a concentration-dependent manner, which is associated with reduced plant growth as a result of an imbalance in ion homeostasis [59]. The differential protein expression in spinach reported altered leaf proteome as a defensive mechanism under Cd stress [60]. Metal stress is believed to accelerate protein and starch degradation by increasing the levels of soluble sugars and amino acids [61] that also support our results. The findings of the present study showed that Cd toxicity probably damages the plasma membrane of root cells thereby altering its permeability which affects nutrient uptake by roots. Plants exposed to Cd stress are responsible for altering the nutrient metabolic balances in roots [62]. Current results demonstrated promotion in various antioxidant enzymes in Cd stressed seedlings of all genotypes, supported by other researchers as well [63]. Enhanced activities of APX, GPX and CAT were also reported earlier in Cd resistant genotypes of many cereals, vegetables and legumes [64–66]. An increase in these enzymes activity under Cd treatment suggested their role in minimizing increased levels of lipid peroxidation and peroxide content caused by ROS, helping the seedlings to maintain their normal growth. Most of the work reported showed an increase in antioxidant enzymes activities with few contrary results. Therefore, the exact mechanisms to scavenge excess hydrogen peroxide levels are not only dependent on plant species but also on some external factors, although at least one of these enzymes is found to be in higher levels under stress conditions. Seeds pretreated with phytohormone prior to Cd reduce APX, CAT, and SOD activity, indicating that the toxic effects have probably overcome efficiently by the antioxidant system. An increase in the level of antioxidative defense enzymes can be considered as their defensive potential under stress. Under severe stress, this system failed to reduce the harmful effects more effectively in a few of our genotypes (NM 2006, NM 13–1 & NM-51), or this could be due to their sensitive genotypes itself. Various cytological irregularities were observed during Cd stress in *Nigella sativa* while post-treatment of SA resulted in a significant increase in MI% with the reduction in chromosomal aberrations. These results are consistent with our findings that observed increase mitotic index and reduced chromosomal aberrations which confirmed ameliorating property of SA under stress [67]. It was reported that Cd tends to reduce the mitotic activity of root cells thus decrease plant growth by shortening G2 phases and prophase that facilitates the abnormal spindle formation while SA pretreatment increased the mitotic index.

Pretreatment with a low concentration of SA before Cd treatment lowered the elevated levels of ROS and enhanced the activities of various antioxidant enzymes thus guarding the plants against oxidative explosion [68]. Our results reported a decline in the activities of the antioxidant enzymes with pretreatment of salicylic acid as compared to Cd stress alone by higher than in control supported by work on rice [69]. There is data on the role played by salicylic acid on plants in signaling and alleviating metal injury [70, 71]. Endogenous and exogenous SA is important in mitigating the uptake of Cd ions and protecting plants against heavy metal stress. Promotion of SA was observed in various Cd-treated plants [72–74]. Particularly, an increase in levels of SA in roots during Cd stress in wheat, might be related to the enhancement of the glutathione cycle, so stimulating antioxidant and metal detoxification systems, which promotes Cd tolerance [75]. The mechanisms that alleviated the Cd toxicity include its uptake reduction and supply in plants, the complex formation and sequestration of Cd, the increase of net photosynthesis and the transpiration rates, to scavenge ROS by enhancing antioxidant defense system to protect membrane [76, 77]. It was suggested earlier that SA is capable of facilitating plant growth and reducing/detoxifying Cd toxicity and could be a promising tool in increasing phytoremediation efficiency. Our results showed that APX and CAT activities decrease in pretreated SA samples while SOD and GPX enzymes further increase in SA pretreatment.

Results obtained from the present investigation revealed that exogenous SA ameliorates the toxic effect of Cd. It was also been demonstrated that the effect of SA not only depends upon its nature, whether it has been provided as acid solely (SA) or as a sodium salt (NaSA). Although the mode of treatment is very crucial as different combinations have variable effects even at the same concentrations [78].

Proline has been cited in the literature as a multi-functional molecule that has a role as an osmoprotectant and a radical scavenger [79]. It is also a helpful stress alleviator that tends to improve plant growth, alter enzyme activity and by increasing MI% under Cd stress [16, 80, 81]. However, our results showed that priming with proline under Cd treatment increases mitotic index and decreases chromosomal aberration caused by Cd. Similar findings were for other abiotic stresses on different crops [82]. Like our results, exogenously supplied proline in cultured tobacco Bright Yellow-2 (BY-2) cells mitigates the Cd-induced inhibitory effects on the growth [83]. This is well-established fact that sugars, amino acids including proline play a major protective role in maintaining osmotic potential or direct detoxification of ROS [84, 85]. Proline accumulated during stress episodes is degraded for energy supply which is utilized to drive growth and relieve the stress, hence helps to continue growth under long‐term stress. Exogenously supplied proline tends to alleviate adverse effects of Cd on growth parameters, antioxidative enzymes capacities, and thus by decreasing MDA and H_2_O_2_ contents [86, 87]. In conclusion, there is a great benefit of applying phytohormones and proline prior to Cd treatment that will be helpful in improving metal tolerance of mung bean seedlings.

## Conclusion

This research suggested that priming with an appropriate amount of phytohormones and proline is a very cost-effective and feasible strategy to induce an adaptive response in mung bean under Cd stress that significantly enhances the mitotic index and decreasing the total percentage of chromosomal abnormalities as compared to Cd treatment alone. An increase in all growth attributes including protein were observed with an increased level of antioxidant enzymes and decreased in lipid peroxidation. Among phytohormones and proline, GA_3_ was found to be more promising in mitigating the adverse effects of Cd for all mung bean varieties tested in this research. These findings will be very helpful for farmers to also make use of Cd polluted lands for cultivation of mung beans after pretreatment of seeds with any of the above phytohormones or proline. There is some hope that this mitigating strategy might allow mung bean to grow better even when irrigating with sewage or industrial wastewater. Further research is in progress to understand complete mechanisms that how phytohormones and proline influence other physiological and biological functions in mung bean under Cd stress.

## Supporting information

S1 Fig Percent promotion/inhibition in mitotic index of mung bean grown under Cd and pretreatments of GA_3_, SA and proline prior to Cd. (TIF)

S2 Fig Percent promotion/inhibition in seedling length of mung bean grown under Cd and pretreatments of GA_3_, SA and proline prior to Cd. (TIF)

S3 Fig Percent promotion/inhibition in fresh weight of mung bean grown under Cd and pretreatments of GA_3_, SA and proline prior to Cd. (TIF)

S4 Fig Percent promotion/inhibition in dry weight of mung bean grown under Cd and pretreatments of GA_3_, SA and proline prior to Cd. (TIF)

S5 Fig Percent promotion/inhibition in relative water content of mung bean grown under Cd and pretreatments of GA_3_, SA and proline prior to Cd. (TIF)

S6 Fig Percent promotion/inhibition in APX activity of mung bean grown under Cd and pretreatments of GA_3_, SA and proline prior to Cd. (TIF)

S7 Fig Percent promotion/inhibition in CAT activity of mung bean grown under Cd and pretreatments of GA_3_, SA and proline prior to Cd. (TIF)

S8 Fig Percent promotion/inhibition in GPX activity of mung bean grown under Cd and pretreatments of GA_3_, SA and proline prior to Cd. (TIF)

S9 Fig Percent promotion/inhibition in SOD activities of mung bean grown under Cd and pretreatments of GA_3_, SA and proline prior to Cd. (TIF)

S10 Fig Percent promotion/inhibition in protein contents of mung bean grown under Cd and pretreatments of GA_3_, SA and proline prior to Cd. (TIF)

S11 Fig Percent promotion/inhibition in MDA contents of mung bean grown under Cd and pretreatments of GA_3_, SA and proline prior to Cd. (TIF)

S12 Fig Percent promotion/inhibition in proline contents of mung bean grown under Cd and pretreatments of GA_3_, SA and proline prior to Cd. (TIF)
