# Supplementary figures and images for: Acclimation of cadmium-induced genotoxicity and oxidative stress in mung bean seedlings by priming effect of phytohormones and proline
Source: PLoS One. 2021 Sep 29;16(9):e0257924. doi: 10.1371/journal.pone.0257924 (PMC8480768; doi:10.1371/journal.pone.0257924)

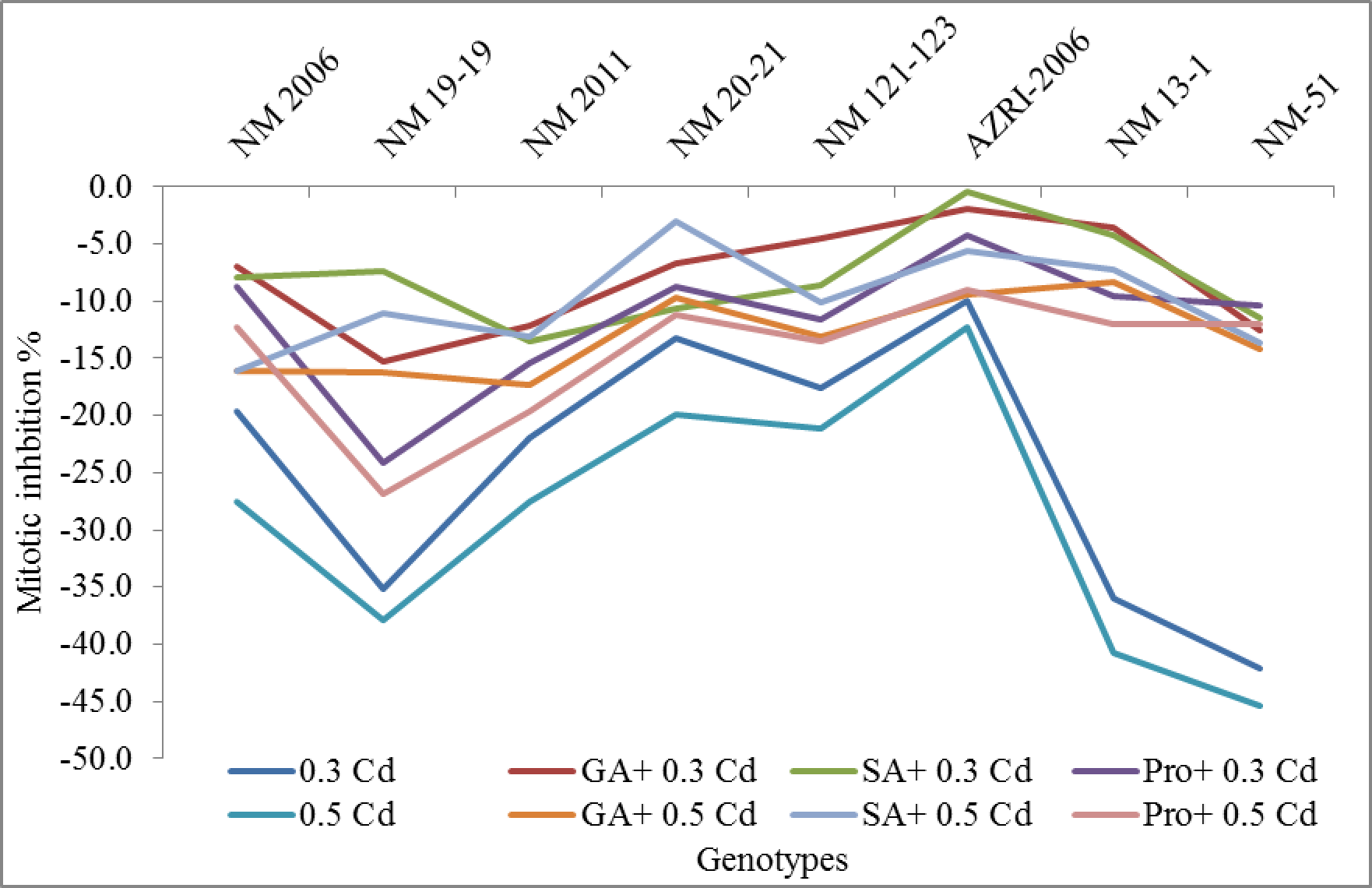

Supplement: S1 Fig — (TIF) [file pone.0257924.s001.tif]

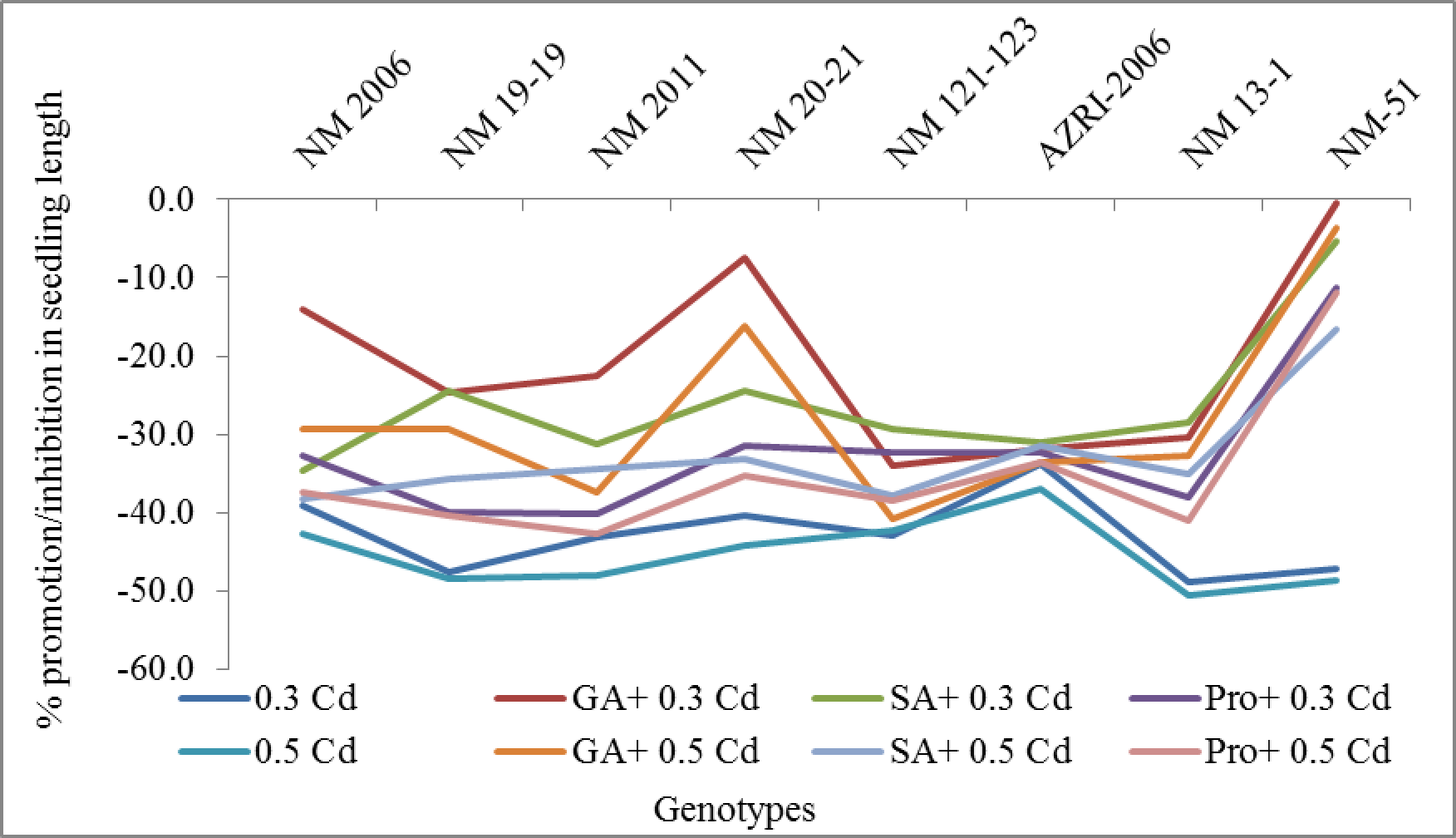

Supplement: S2 Fig — (TIF) [file pone.0257924.s002.tif]

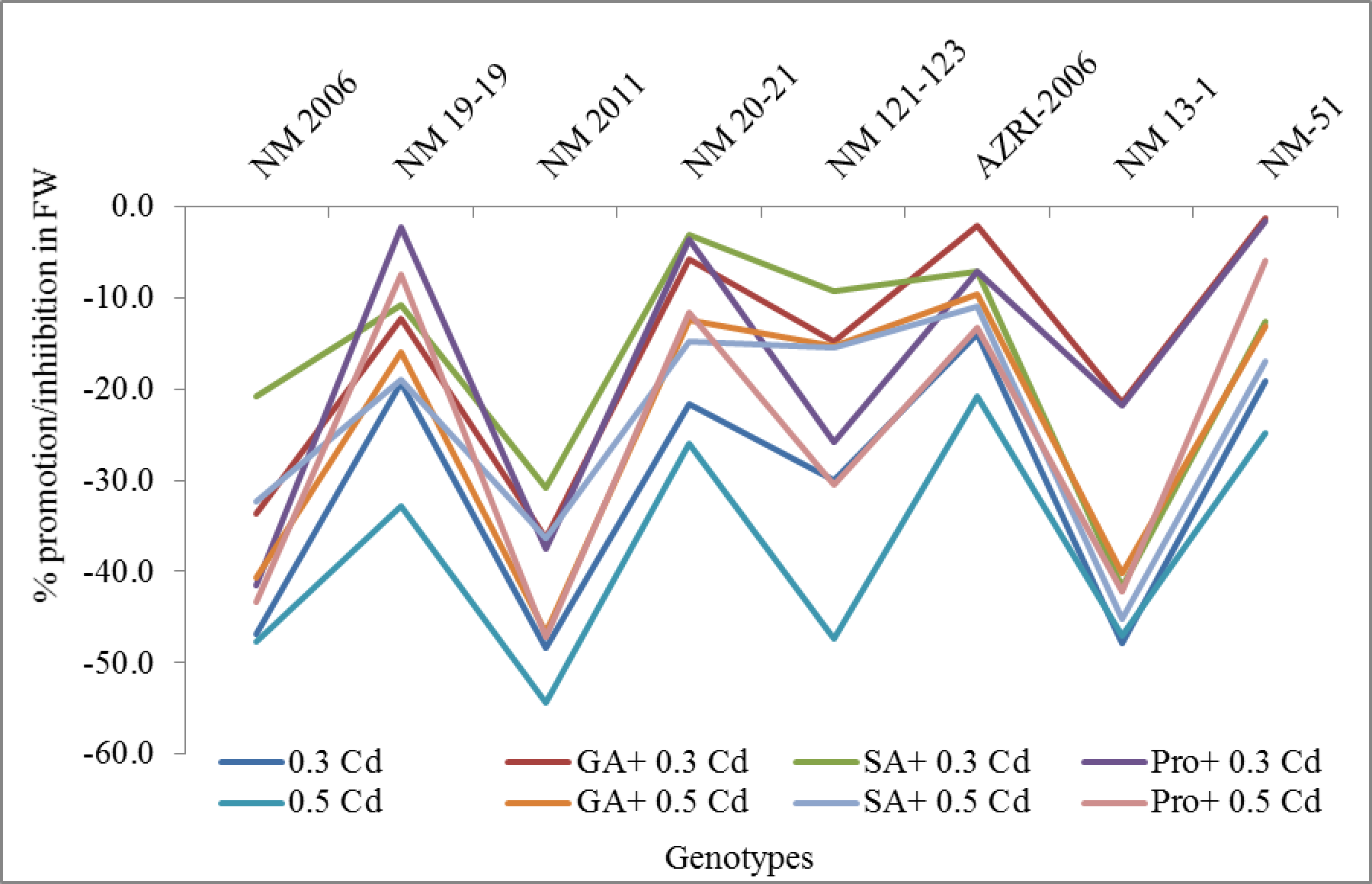

Supplement: S3 Fig — (TIF) [file pone.0257924.s003.tif]

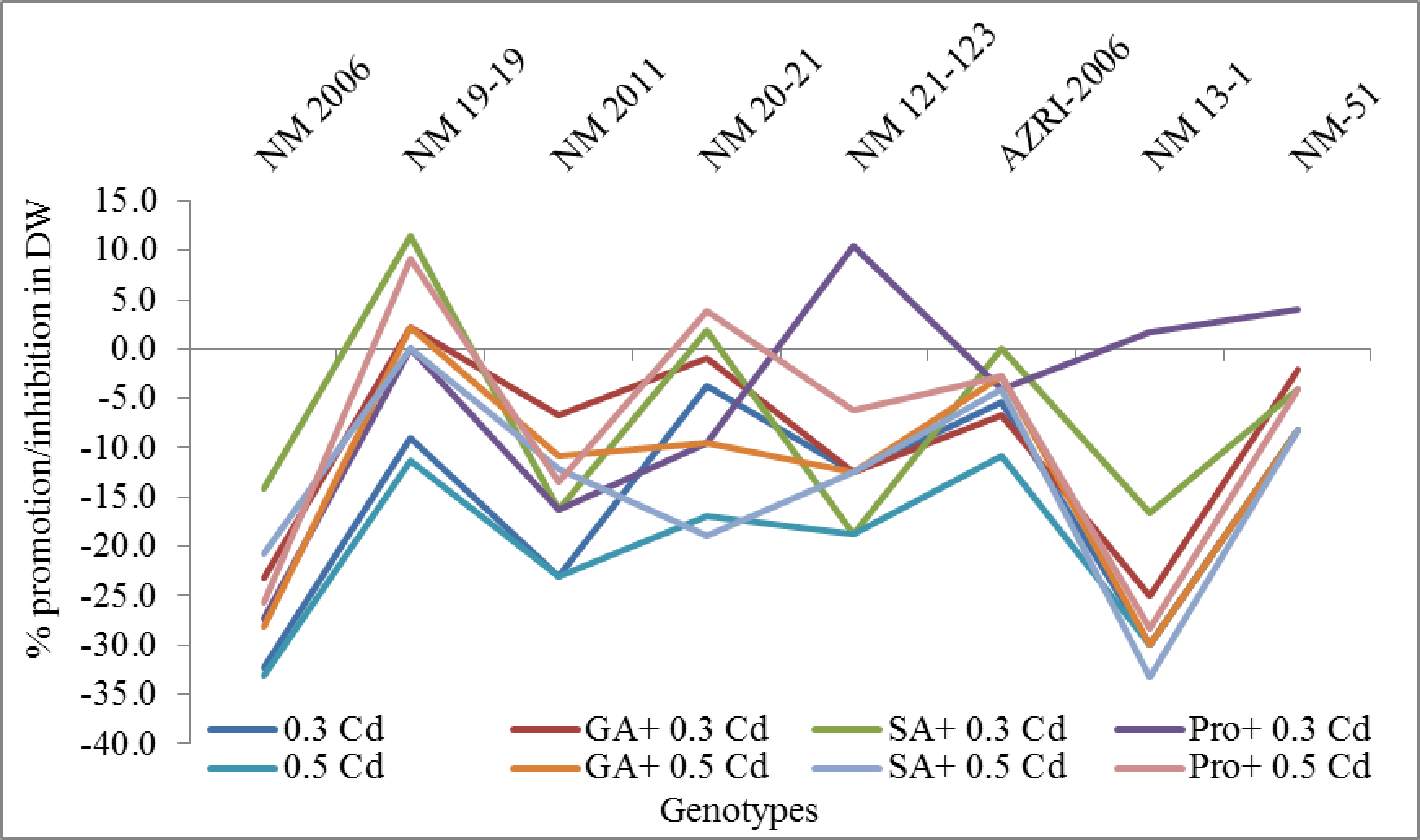

Supplement: S4 Fig — (TIF) [file pone.0257924.s004.tif]

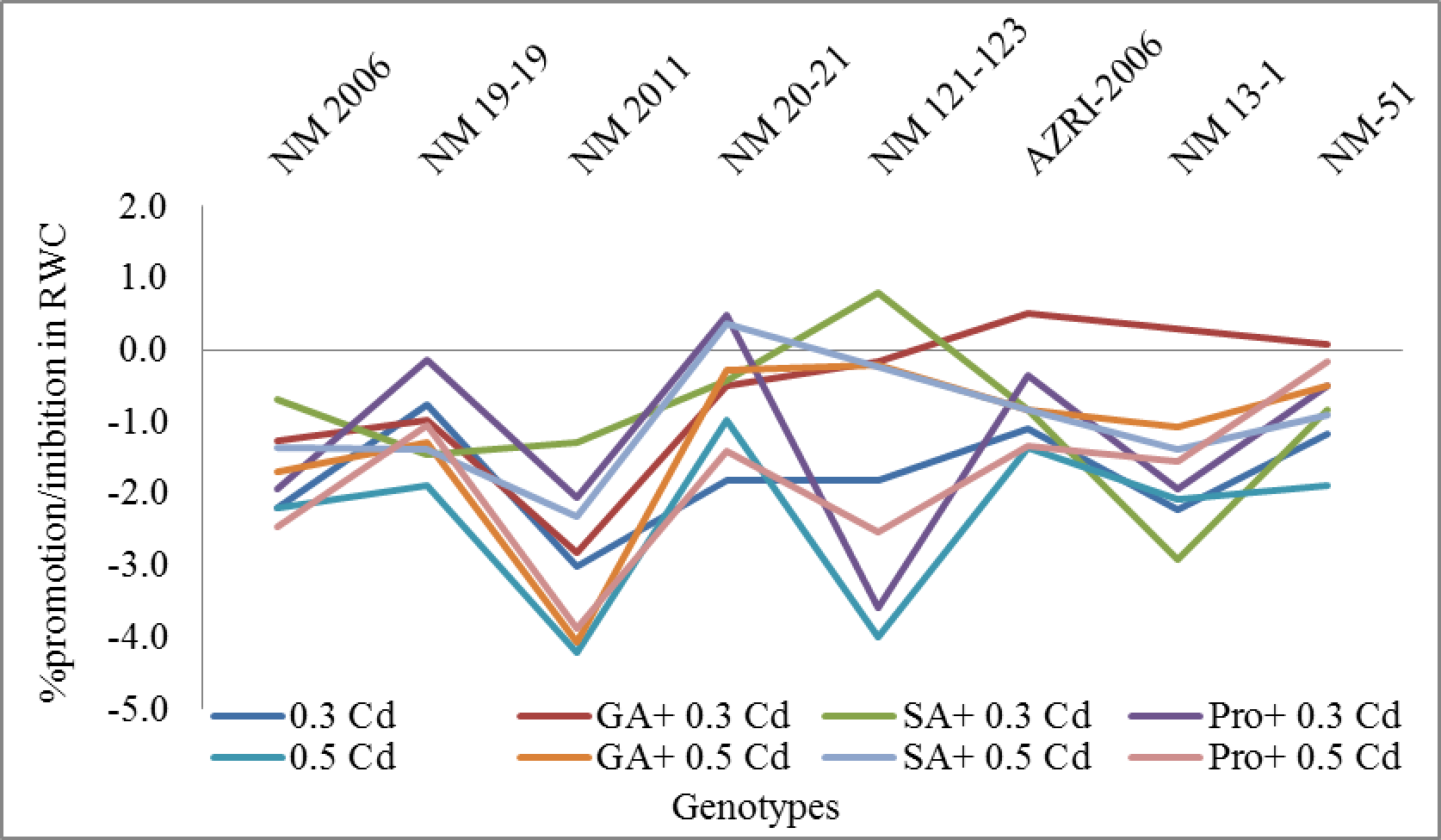

Supplement: S5 Fig — (TIF) [file pone.0257924.s005.tif]

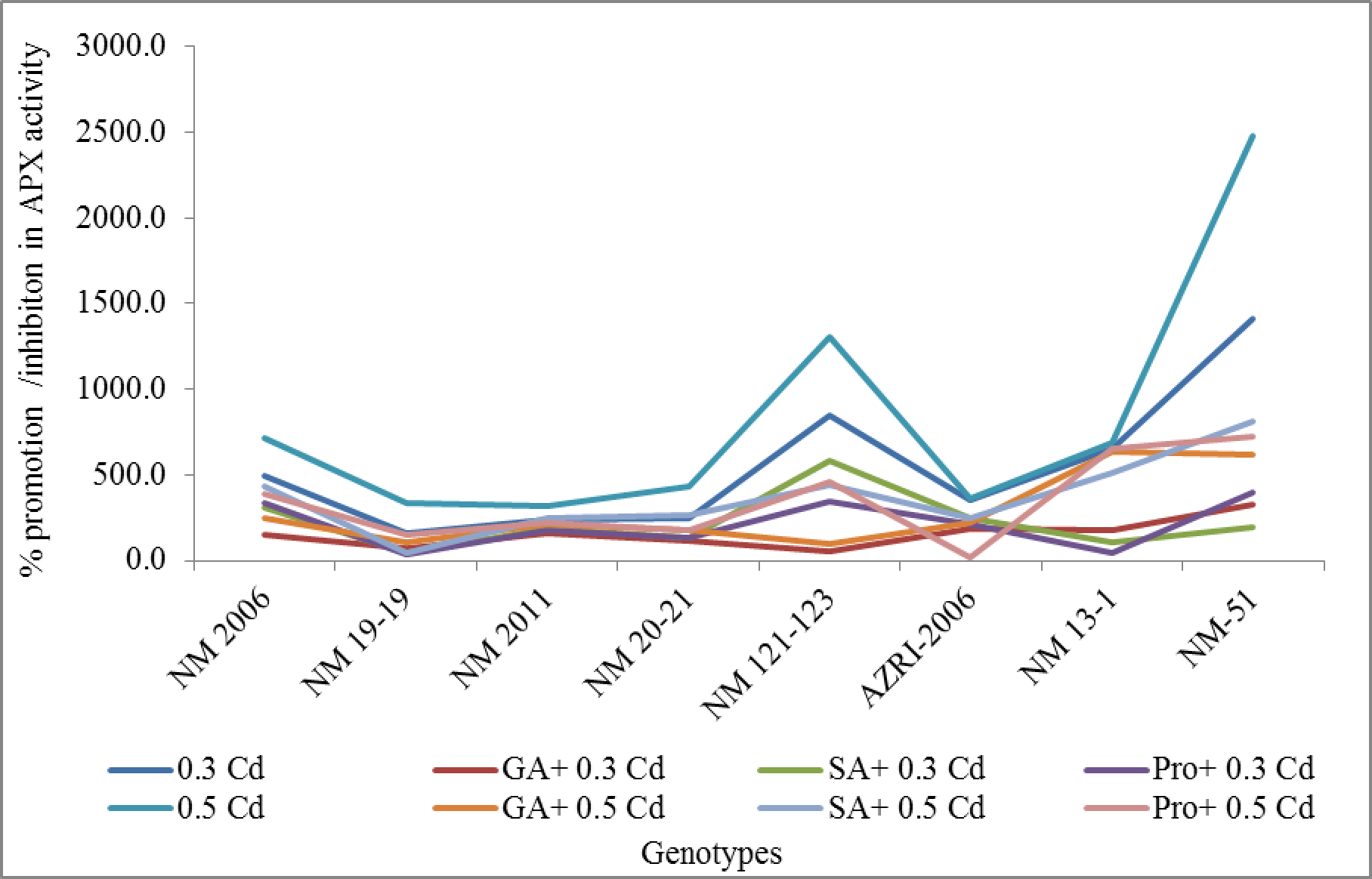

Supplement: S6 Fig — (TIF) [file pone.0257924.s006.tif]

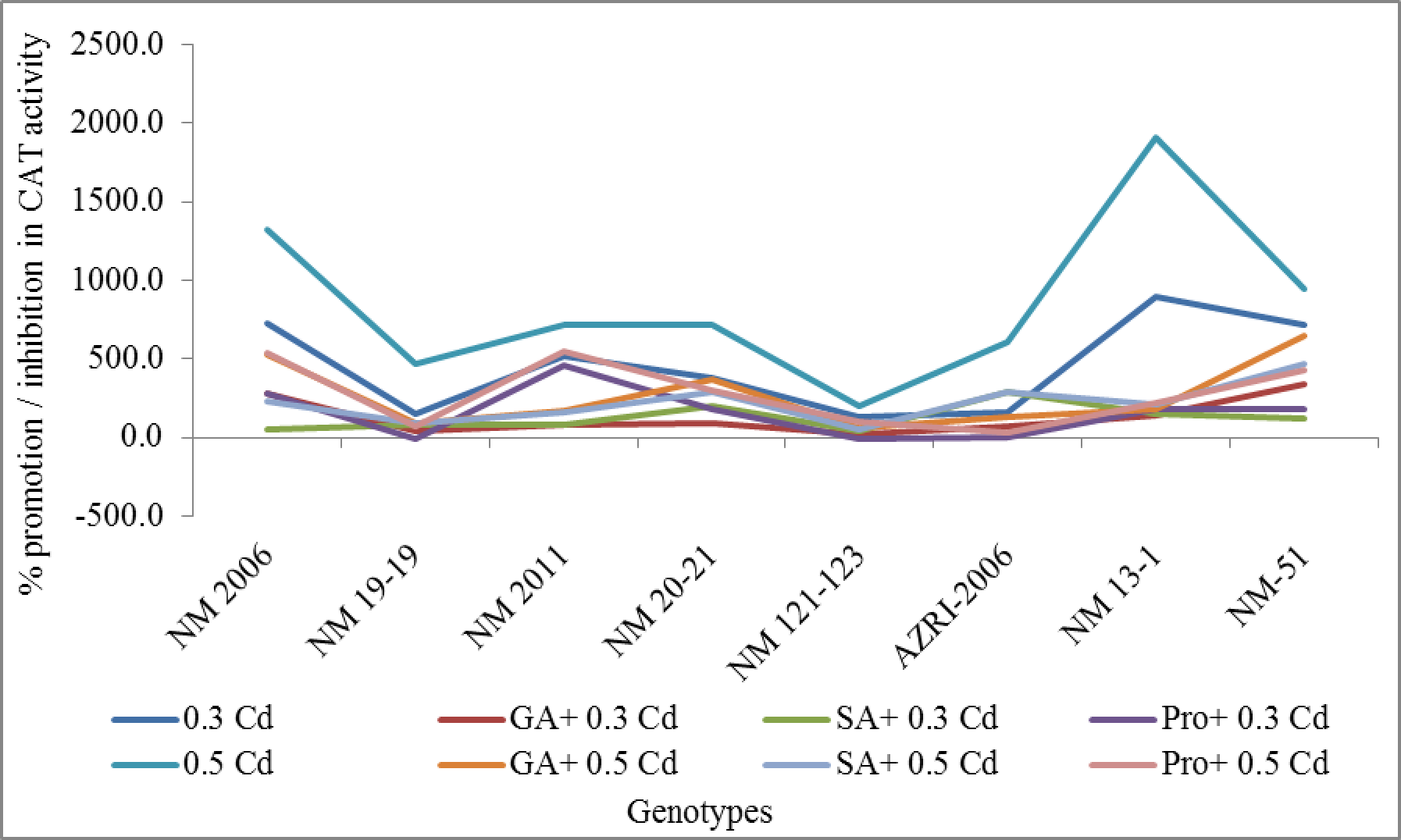

Supplement: S7 Fig — (TIF) [file pone.0257924.s007.tif]

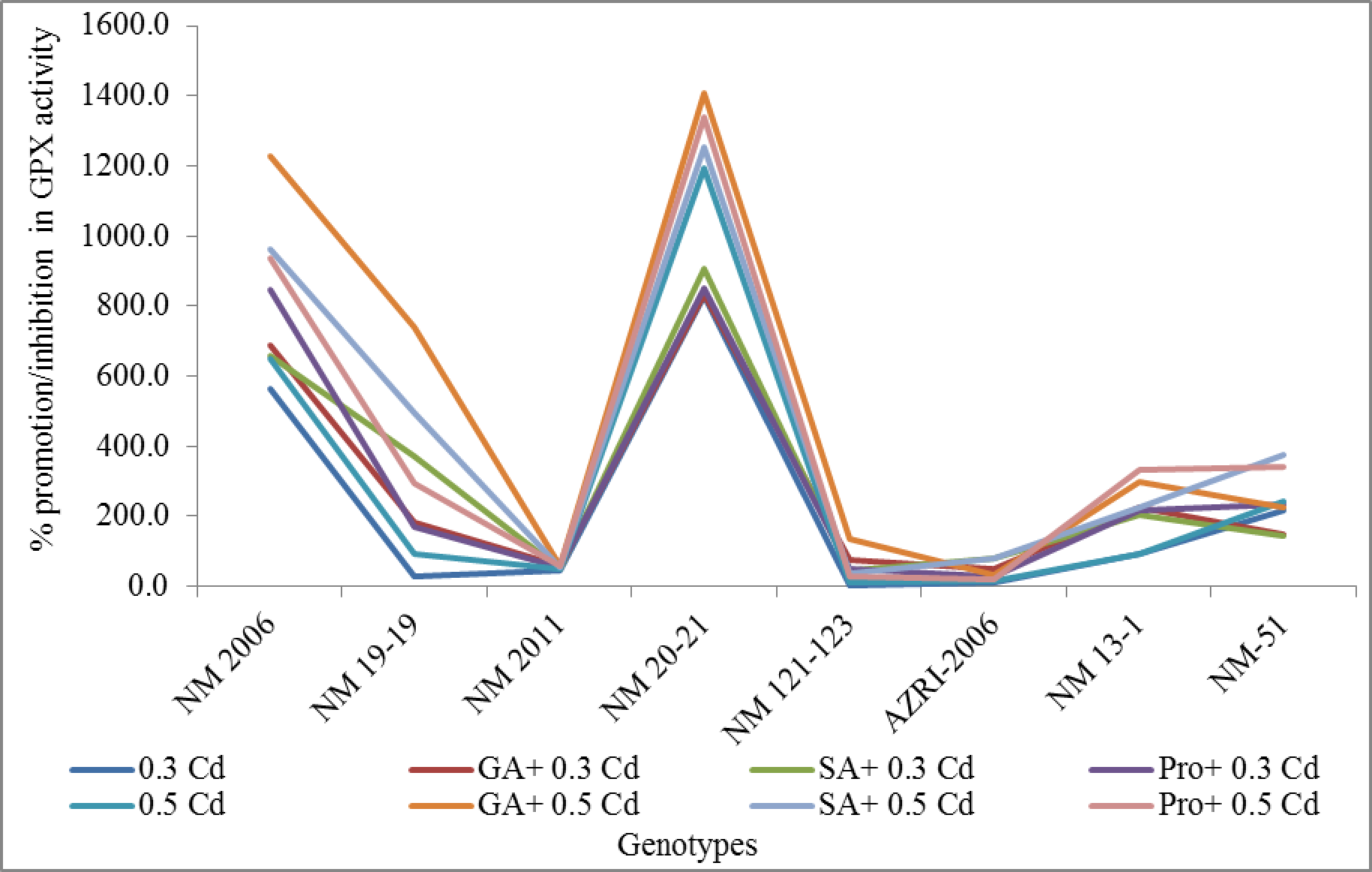

Supplement: S8 Fig — (TIF) [file pone.0257924.s008.tif]

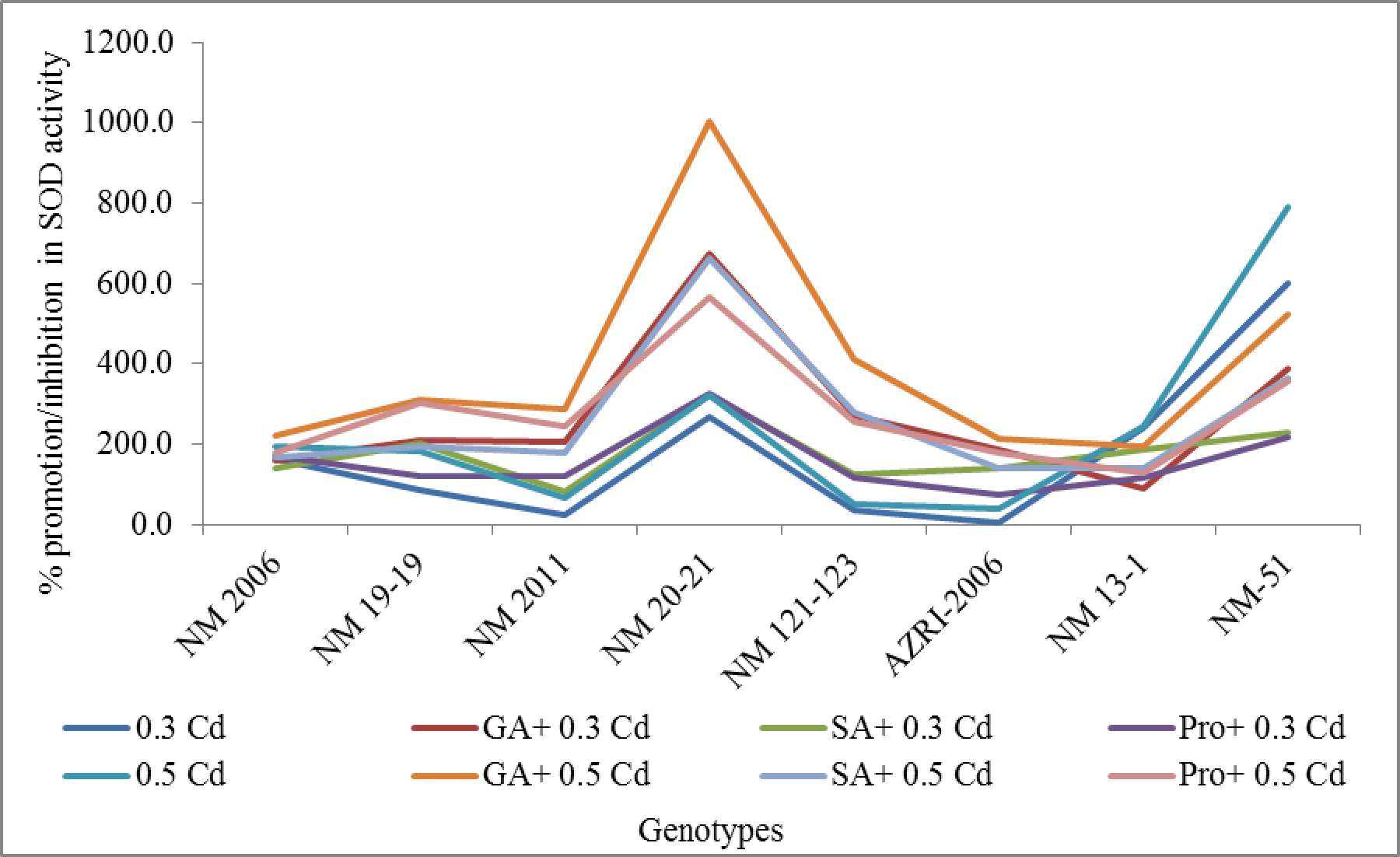

Supplement: S9 Fig — (TIF) [file pone.0257924.s009.tif]

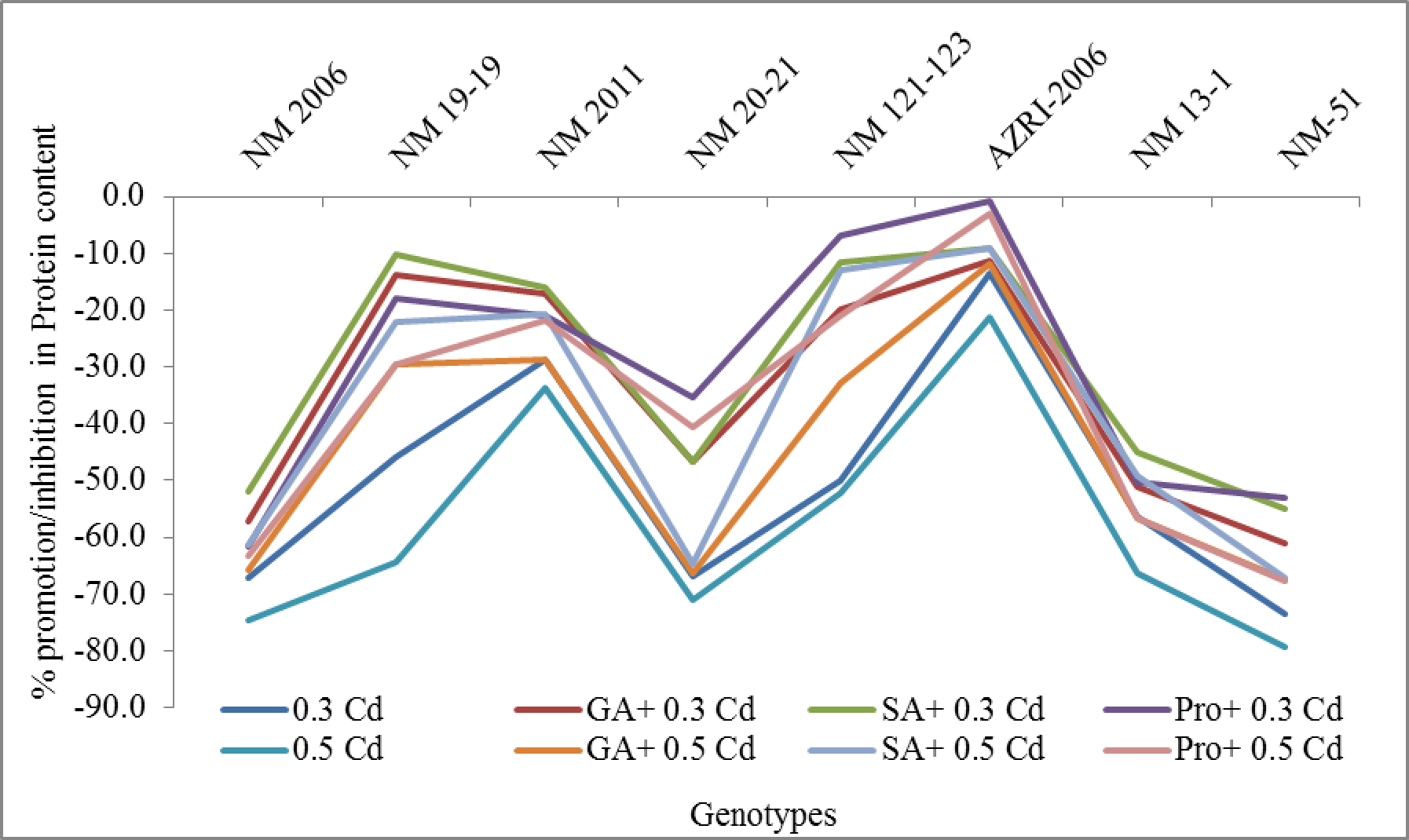

Supplement: S10 Fig — (TIF) [file pone.0257924.s010.tif]

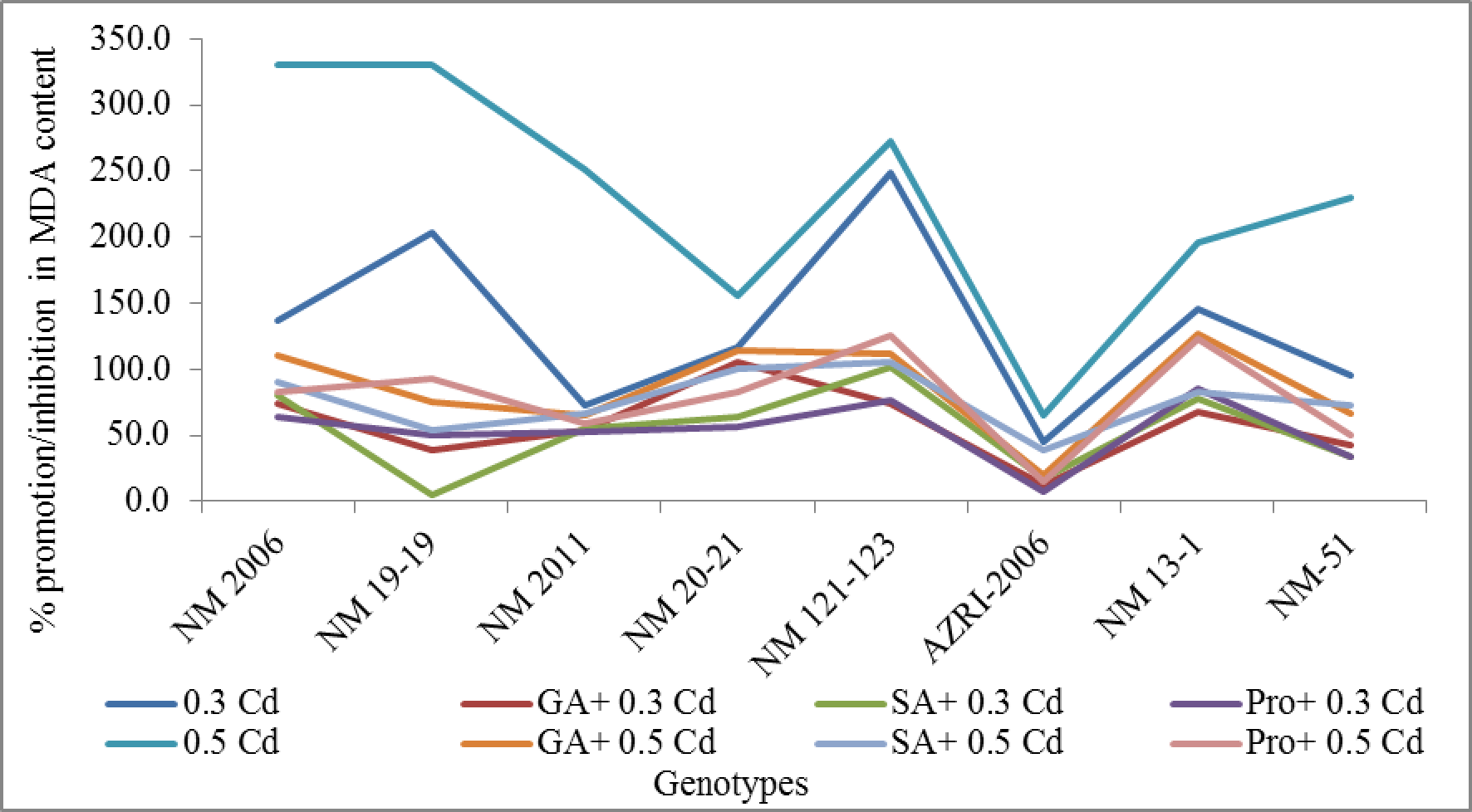

Supplement: S11 Fig — (TIF) [file pone.0257924.s011.tif]

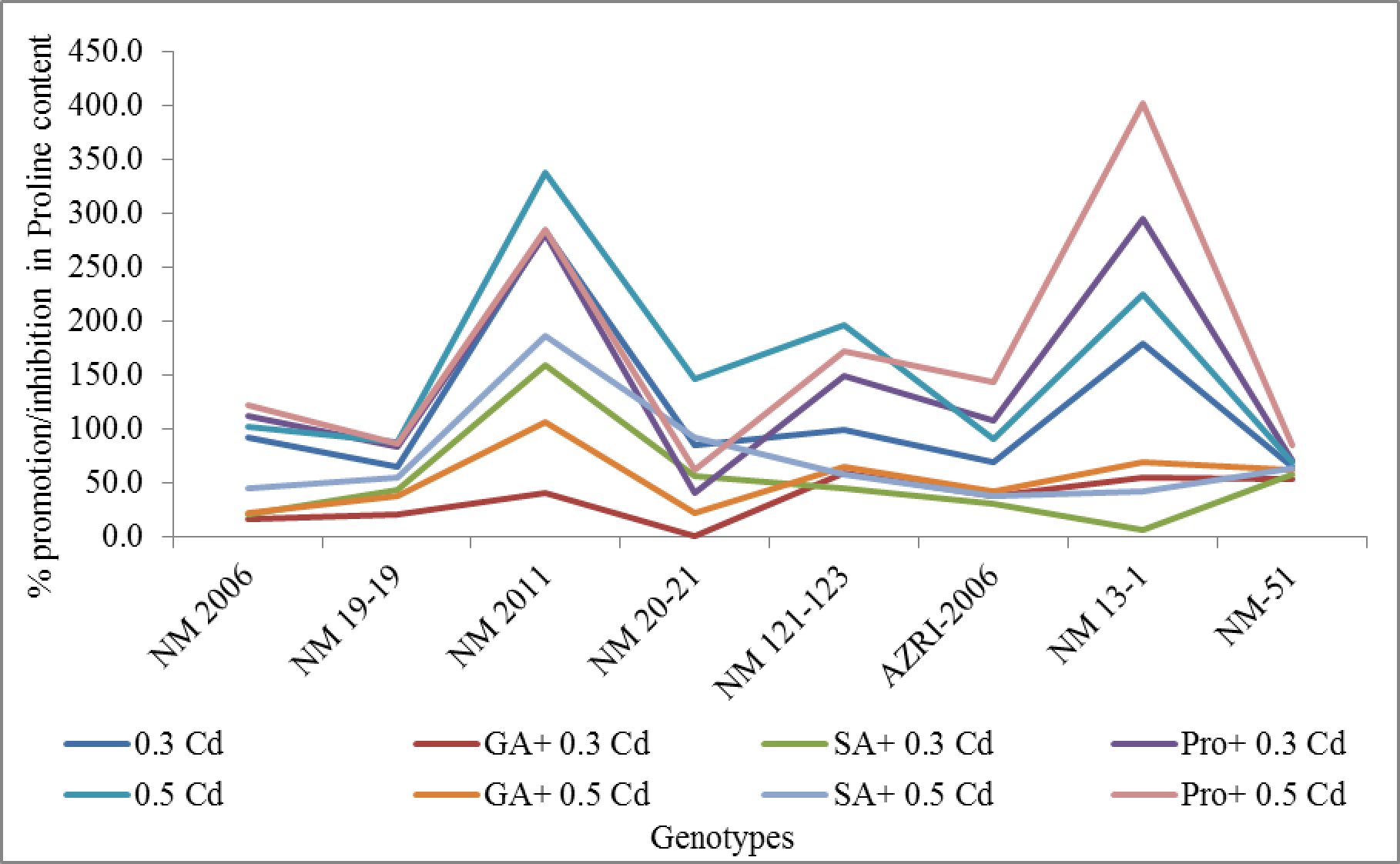

Supplement: S12 Fig — (TIF) [file pone.0257924.s012.tif]
